# Supplementary figures and images for: Atypical peripheral actin band formation via overactivation of RhoA and nonmuscle myosin II in mitofusin 2-deficient cells (part 1 of 2)
Source: eLife. 2023 Sep 19;12:e88828. doi: 10.7554/eLife.88828 (PMC10550287; doi:10.7554/eLife.88828)

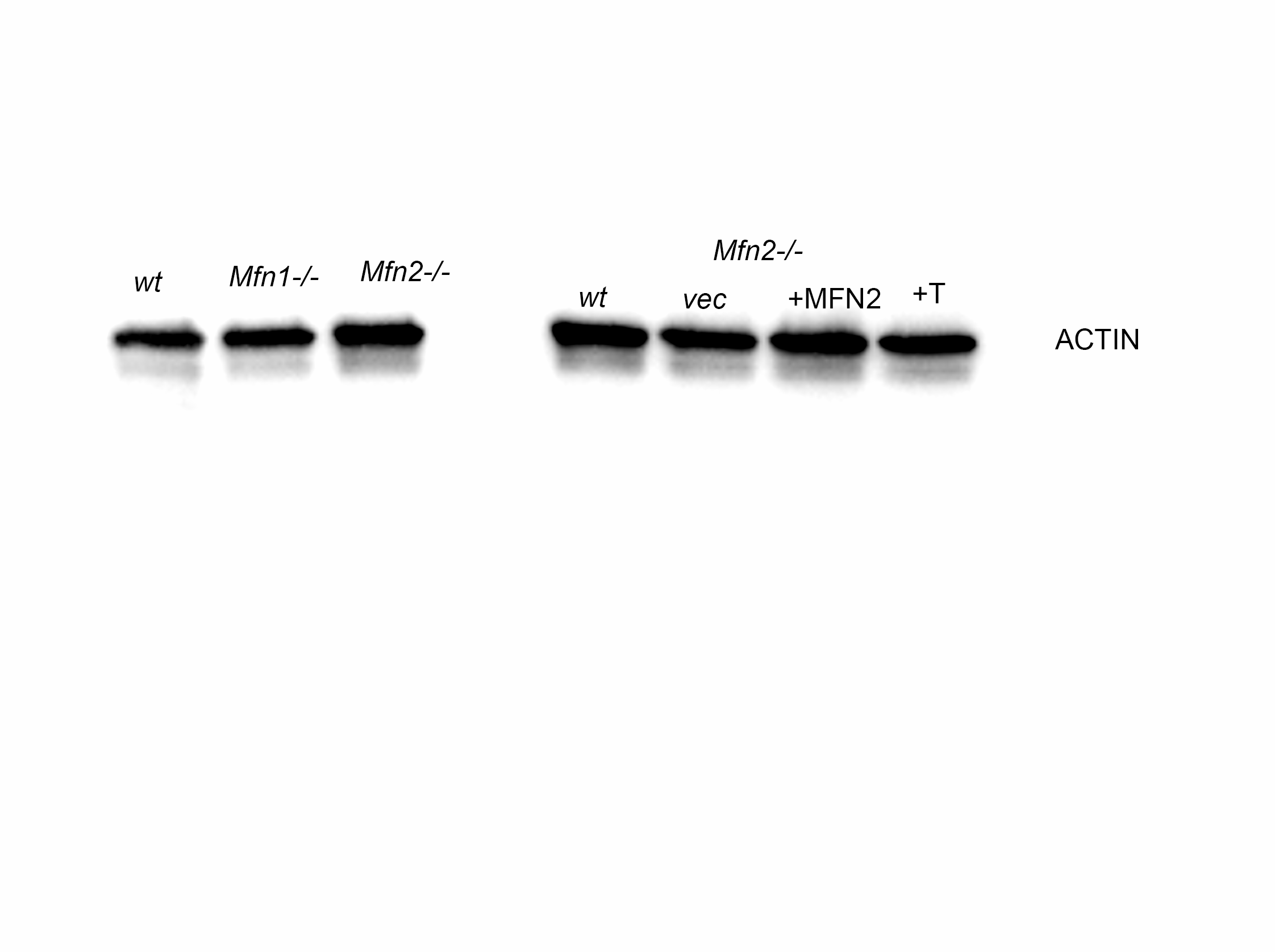

Supplement: Figure 1—source data 1. [file elife-88828-fig1-data1.zip › Figure 1-source data 1/ACTIN/Wang, Yueyang 2023-02-11_13h57m24sMEF ACTIN.tif]

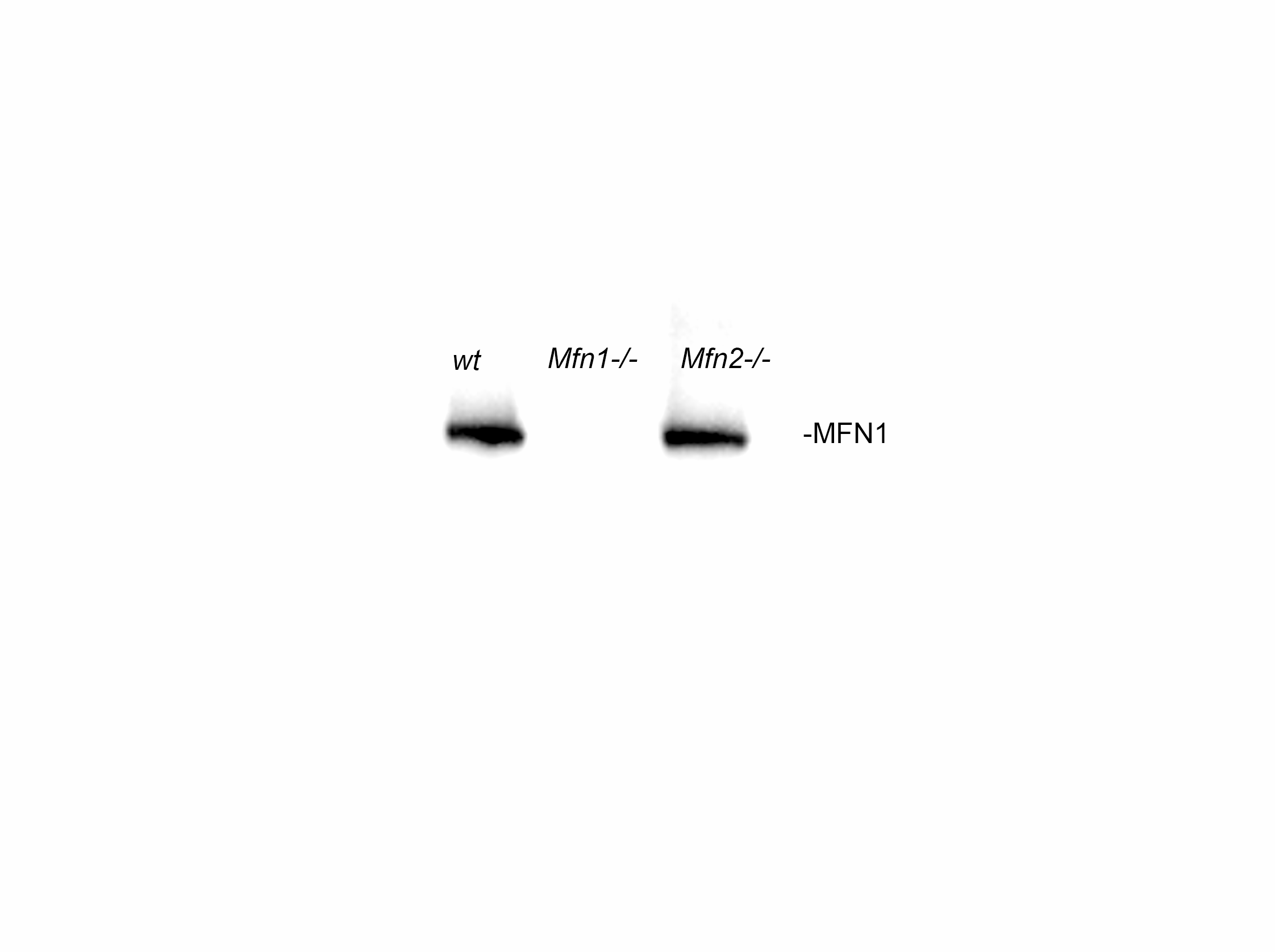

Supplement: Figure 1—source data 1. [file elife-88828-fig1-data1.zip › Figure 1-source data 1/MFN1/Wang, Yueyang 2023-02-11_13h42m13s MEF mfn1.tif]

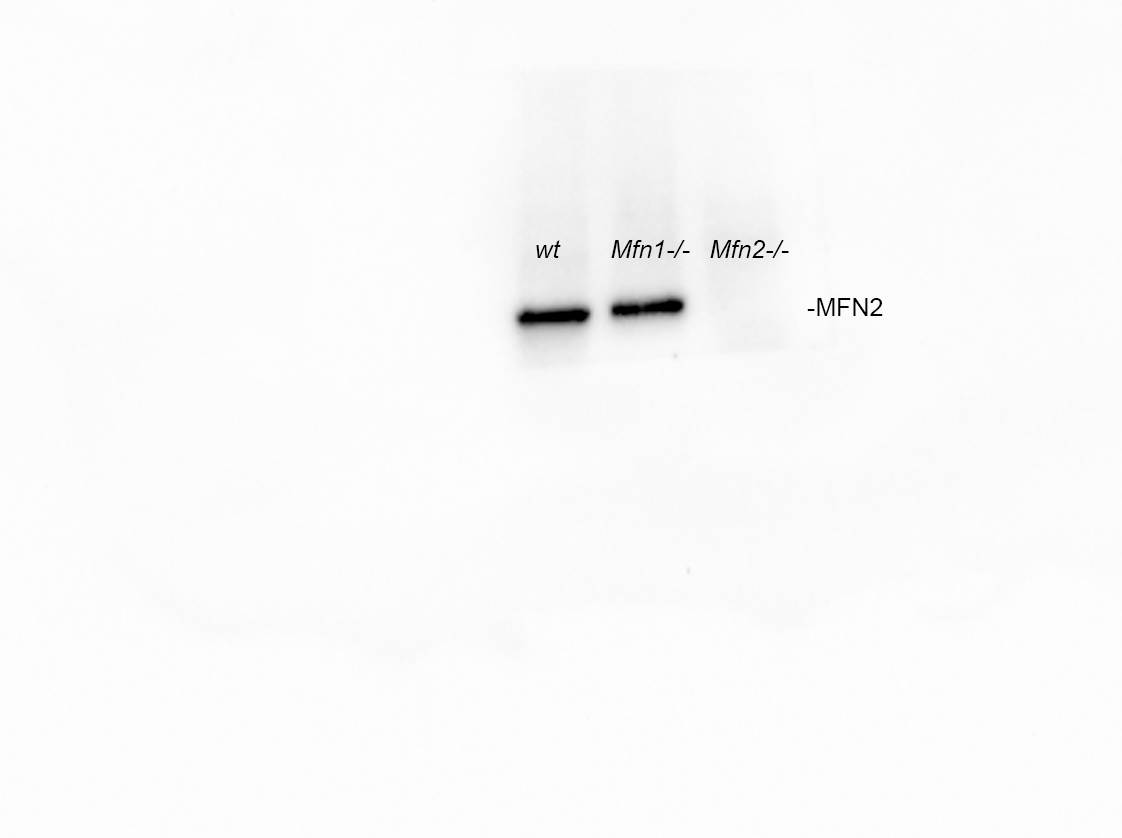

Supplement: Figure 1—source data 1. [file elife-88828-fig1-data1.zip › Figure 1-source data 1/MFN2/Wang, Yueyang 2023-02-01_19h49m55s mfn2.tif]

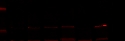

Supplement: Figure 2—source data 1. [file elife-88828-fig2-data1.zip › Figure 2-source data 1/MFN1/0000713_01_TH.jpg]

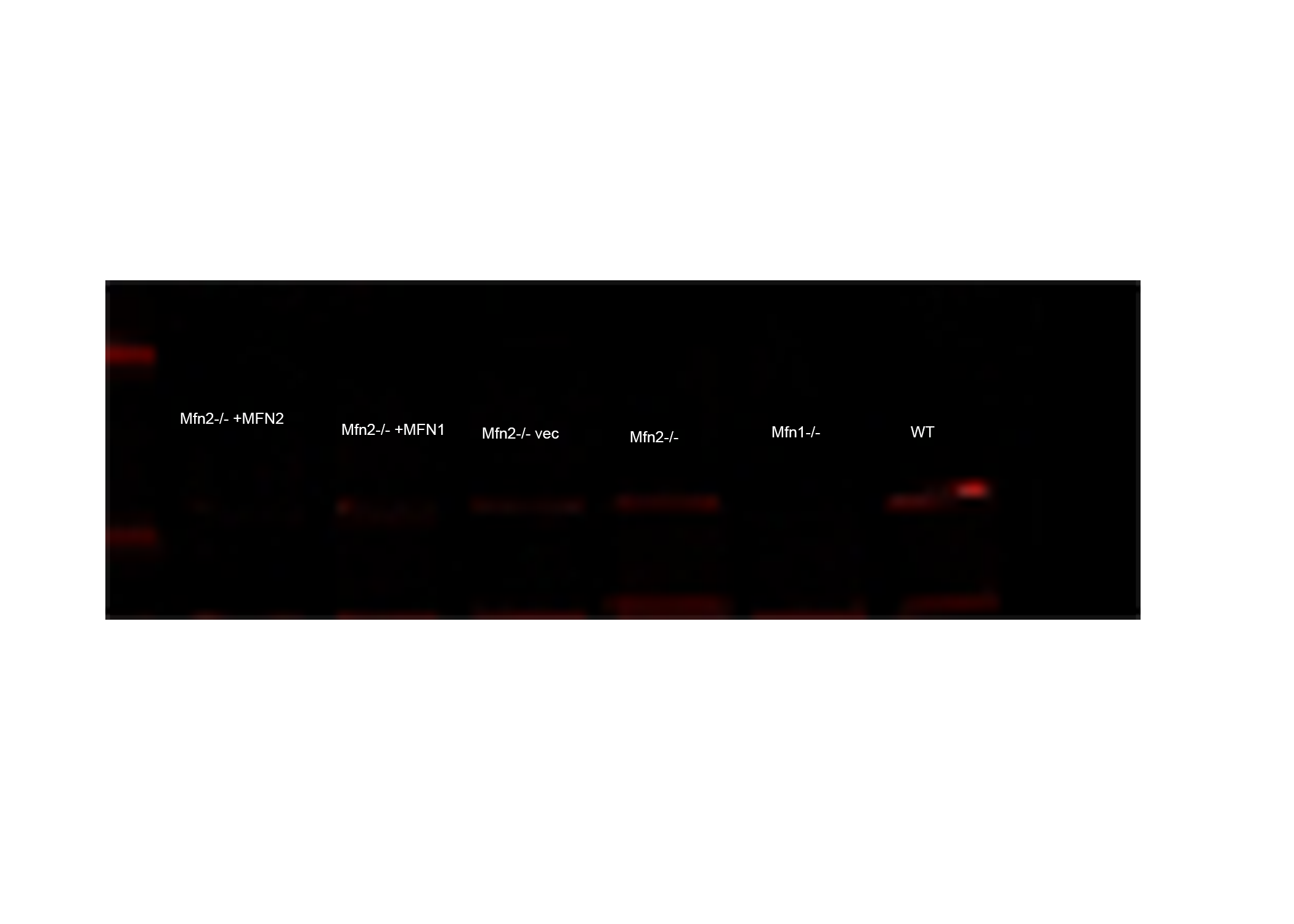

Supplement: Figure 2—source data 1. [file elife-88828-fig2-data1.zip › Figure 2-source data 1/MFN1/label.tif]

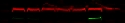

Supplement: Figure 2—source data 1. [file elife-88828-fig2-data1.zip › Figure 2-source data 1/Mfn2 tubulin2/0000708_01/0000708_01_TH.jpg]

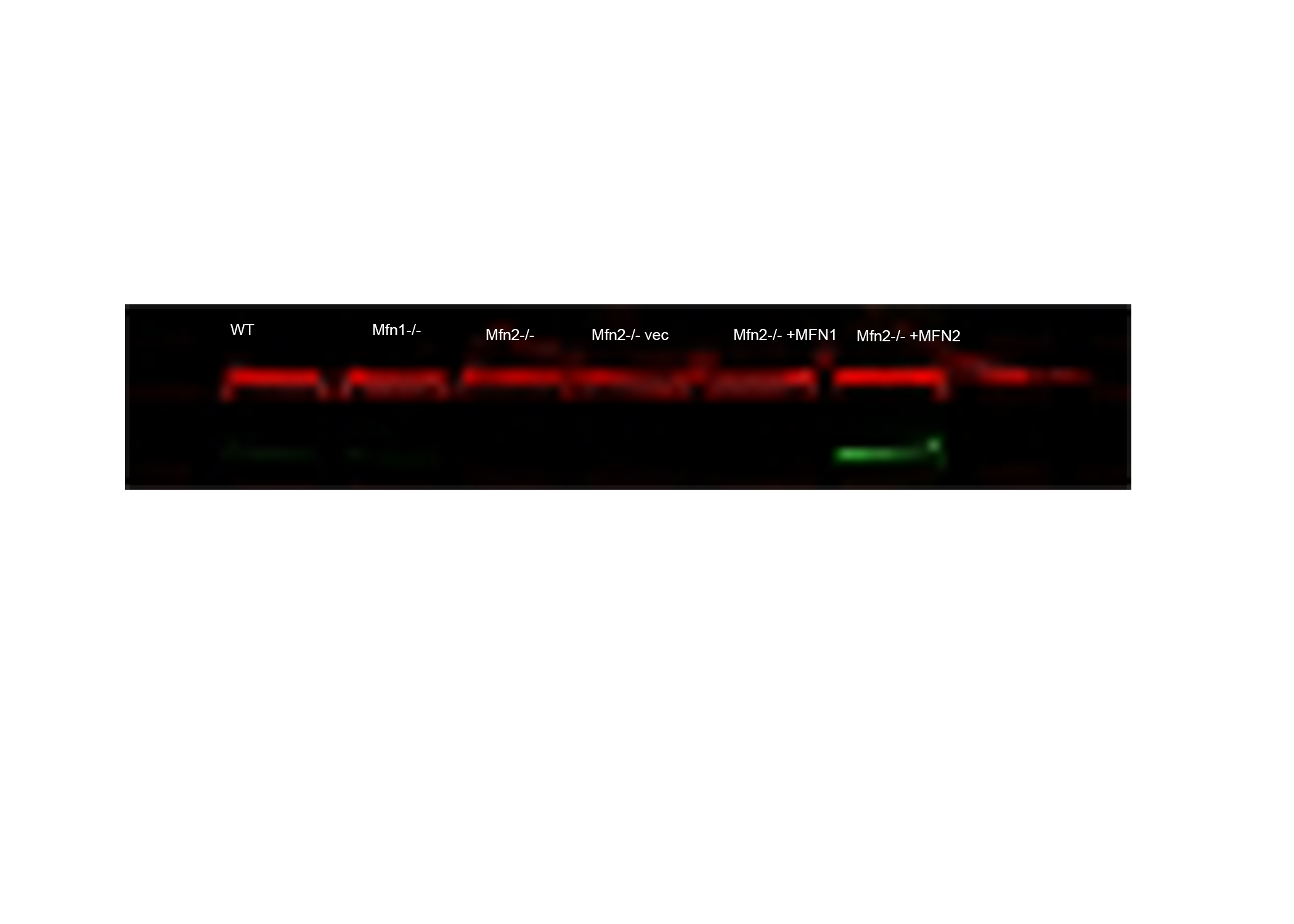

Supplement: Figure 2—source data 1. [file elife-88828-fig2-data1.zip › Figure 2-source data 1/Mfn2 tubulin2/0000708_01/label.tif]

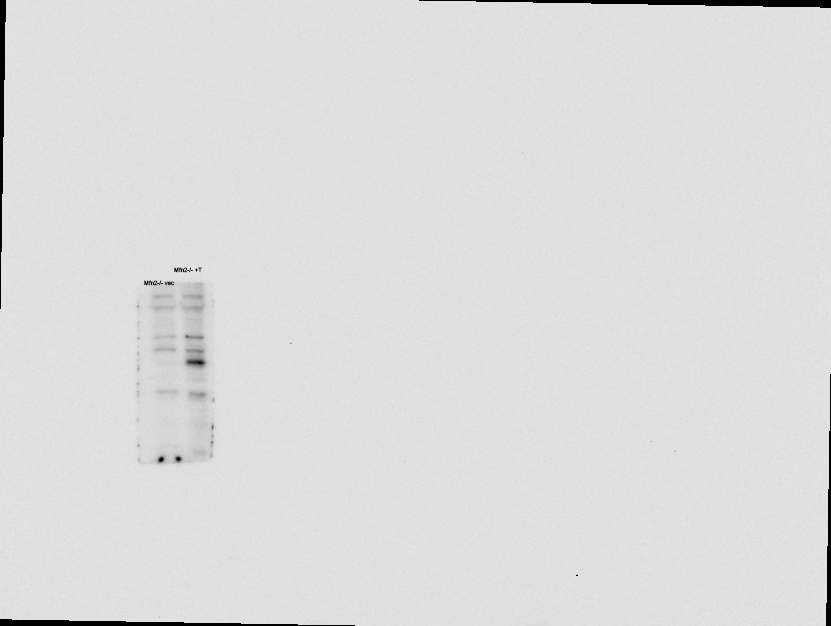

Supplement: Figure 3—source data 1. [file elife-88828-fig3-data1.zip › Figure 3-source data 1/GFP/Gfp_1_16bit.label.tif]

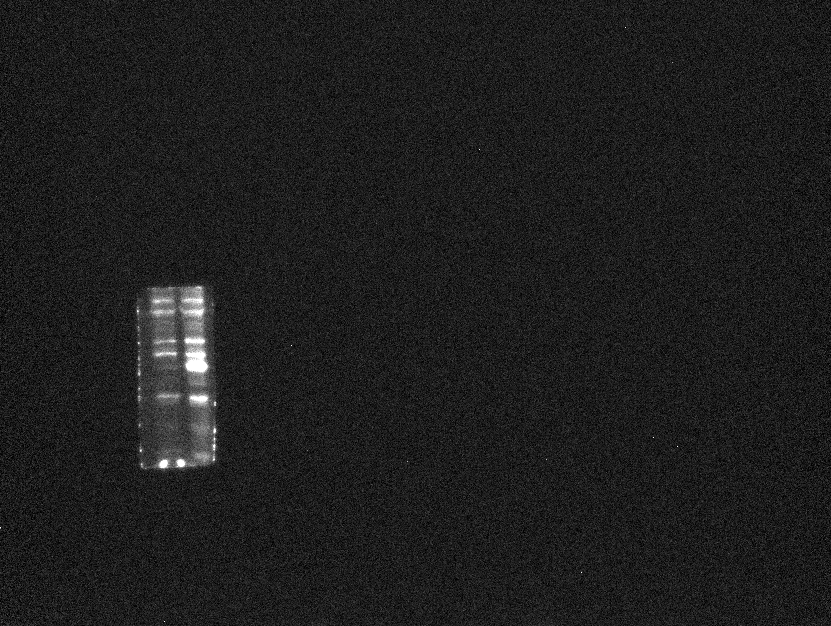

Supplement: Figure 3—source data 1. [file elife-88828-fig3-data1.zip › Figure 3-source data 1/GFP/Gfp_1_16bit.png]

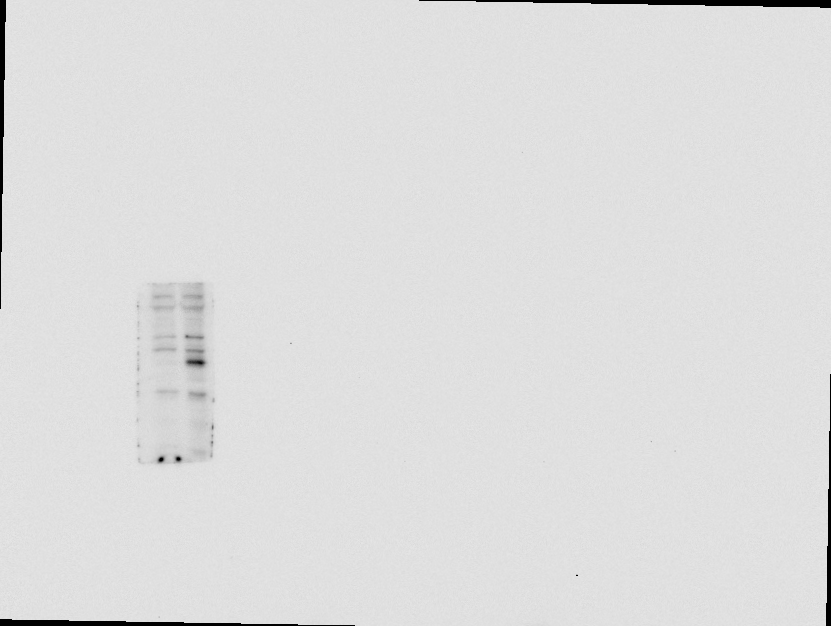

Supplement: Figure 3—source data 1. [file elife-88828-fig3-data1.zip › Figure 3-source data 1/GFP/Gfp_1_16bit.tif-Deuteranope.tif]

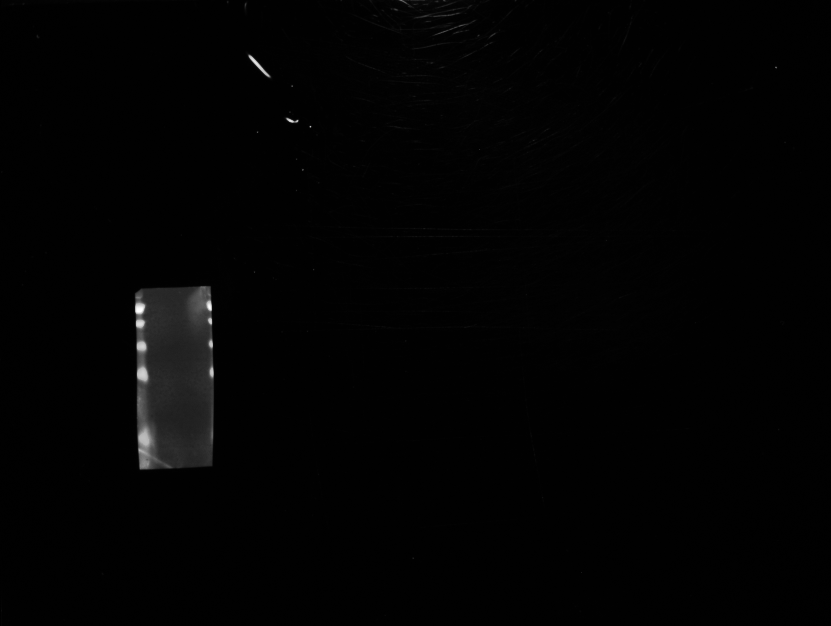

Supplement: Figure 3—source data 1. [file elife-88828-fig3-data1.zip › Figure 3-source data 1/GFP/Gfp_2_16bit.png]

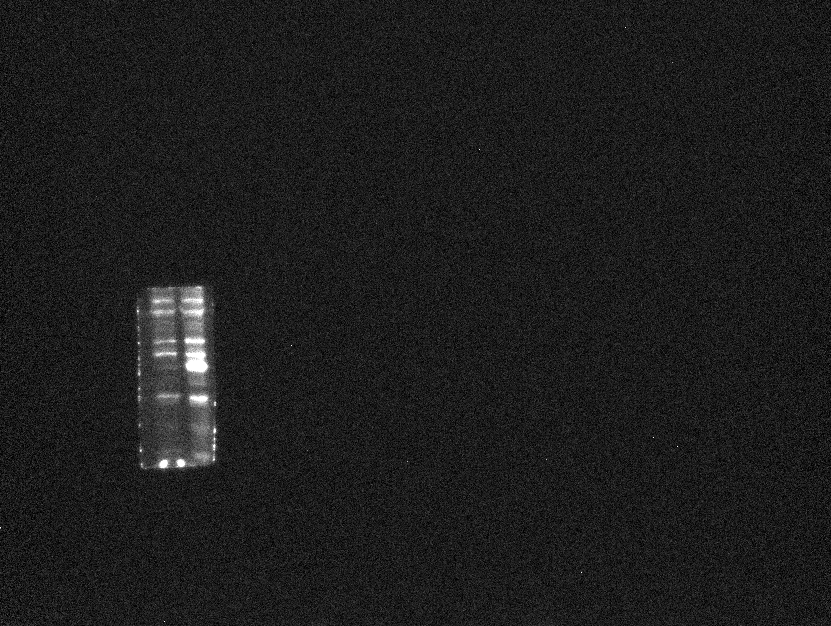

Supplement: Figure 3—source data 1. [file elife-88828-fig3-data1.zip › Figure 3-source data 1/GFP/Gfp_8bit.png]

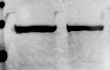

Supplement: Figure 3—source data 1. [file elife-88828-fig3-data1.zip › Figure 3-source data 1/VINCULIn/0000714_01_700VinculinTIF-Deuteranope.tif]

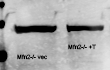

Supplement: Figure 3—source data 1. [file elife-88828-fig3-data1.zip › Figure 3-source data 1/VINCULIn/0000714_01_700VinculinTIF-label.tif]

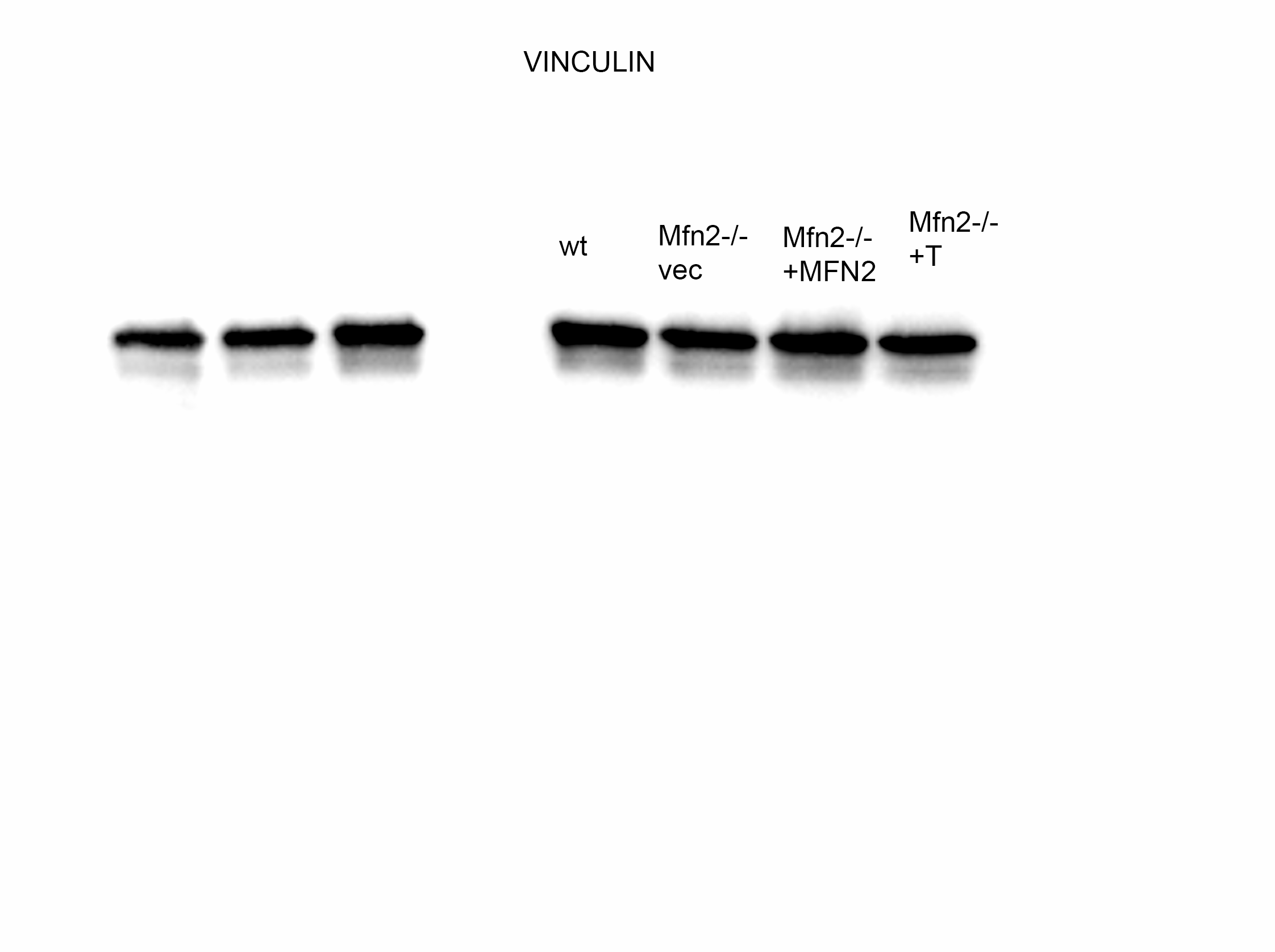

Supplement: Figure 4—source data 1. [file elife-88828-fig4-data1.zip › Figure 4-source data 1/ACTIN/Wang, Yueyang 2023-02-11_13h57m24sMEF ACTIN label.tif]

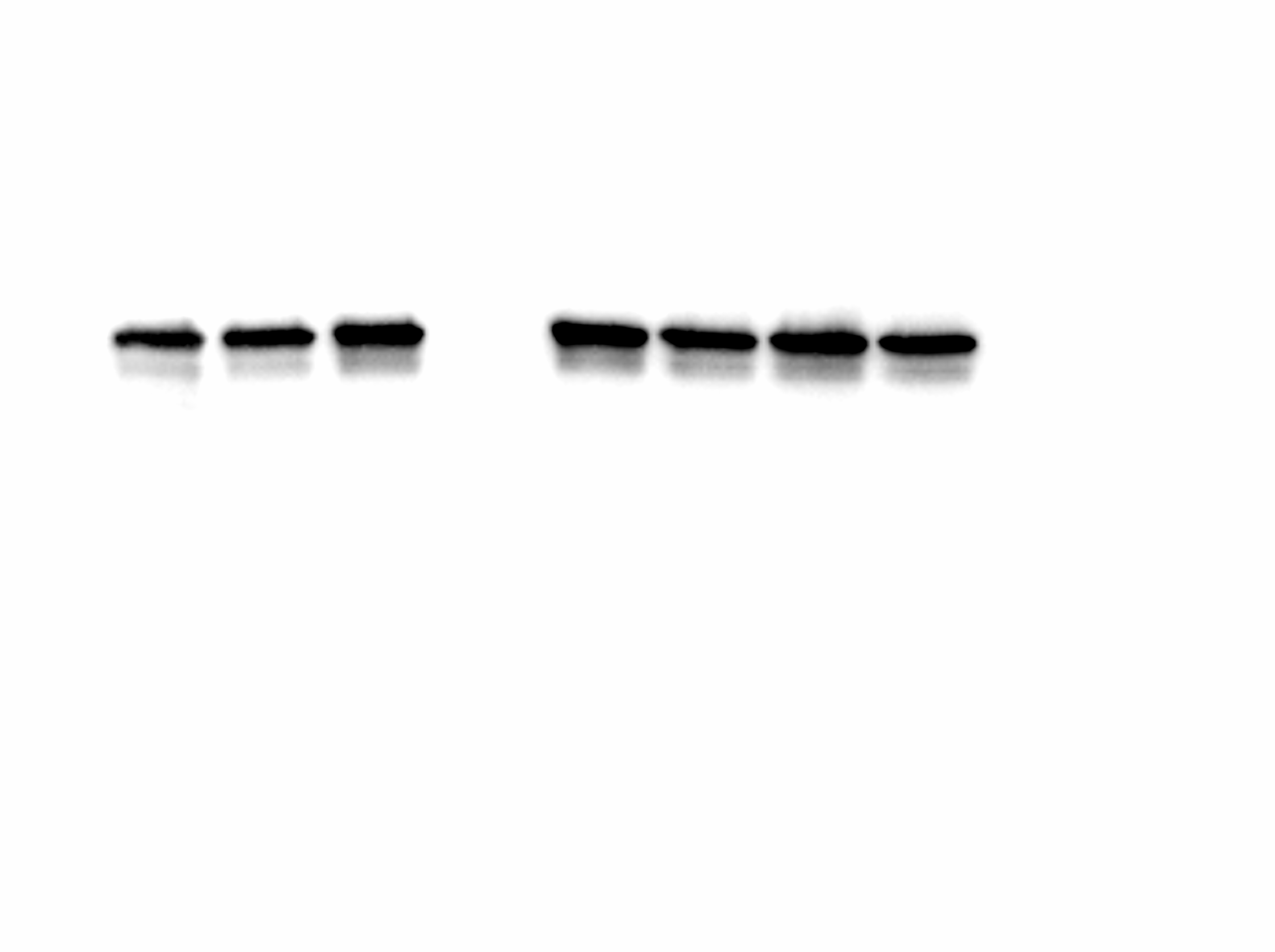

Supplement: Figure 4—source data 1. [file elife-88828-fig4-data1.zip › Figure 4-source data 1/ACTIN/Wang, Yueyang 2023-02-11_13h57m24sMEF ACTIN.tif]

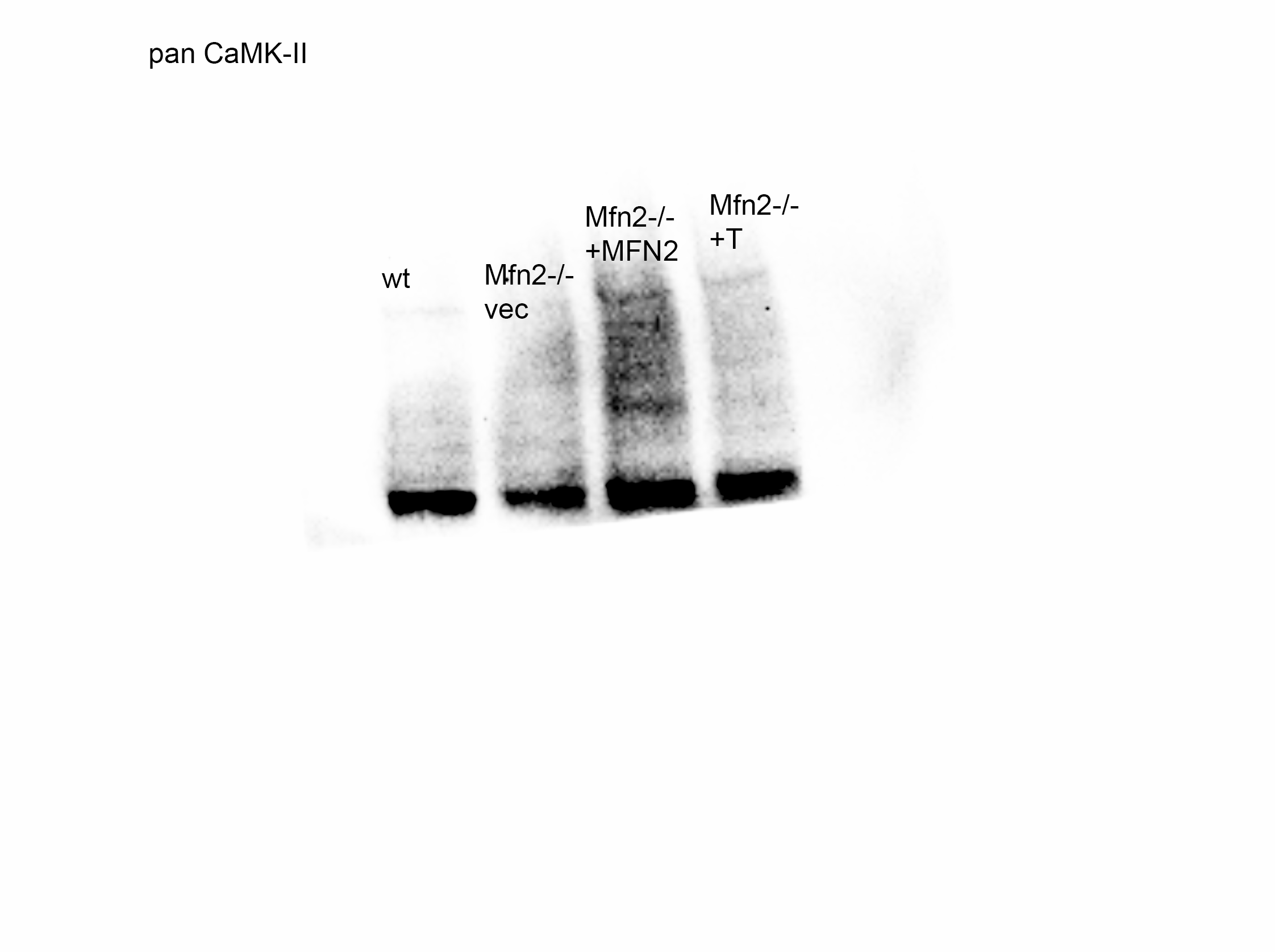

Supplement: Figure 4—source data 1. [file elife-88828-fig4-data1.zip › Figure 4-source data 1/pan CaMKII/Wang, Yueyang 2023-02-11_13h51m47s camkii pan label.tif]

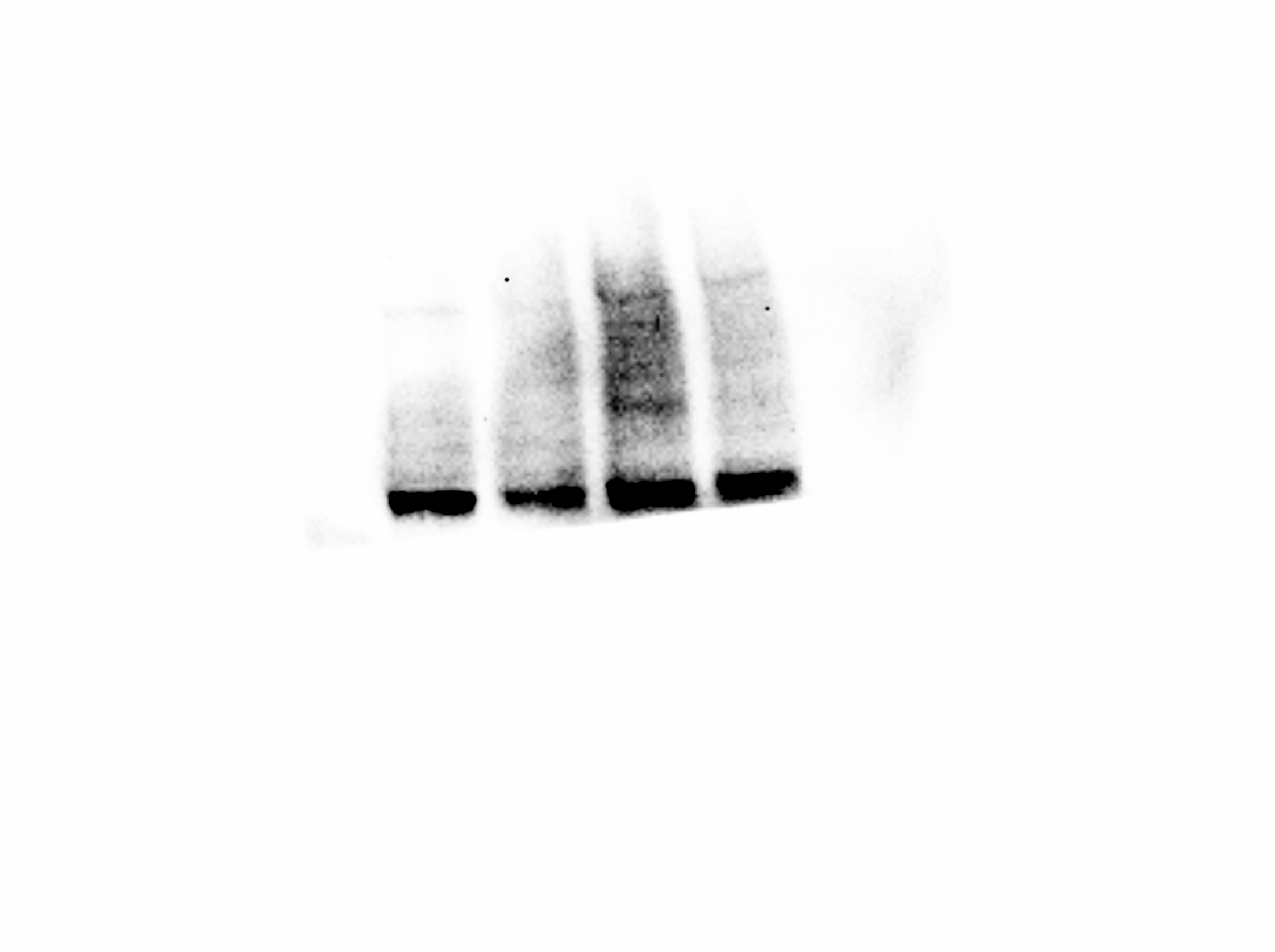

Supplement: Figure 4—source data 1. [file elife-88828-fig4-data1.zip › Figure 4-source data 1/pan CaMKII/Wang, Yueyang 2023-02-11_13h51m47s camkii pan.tif]

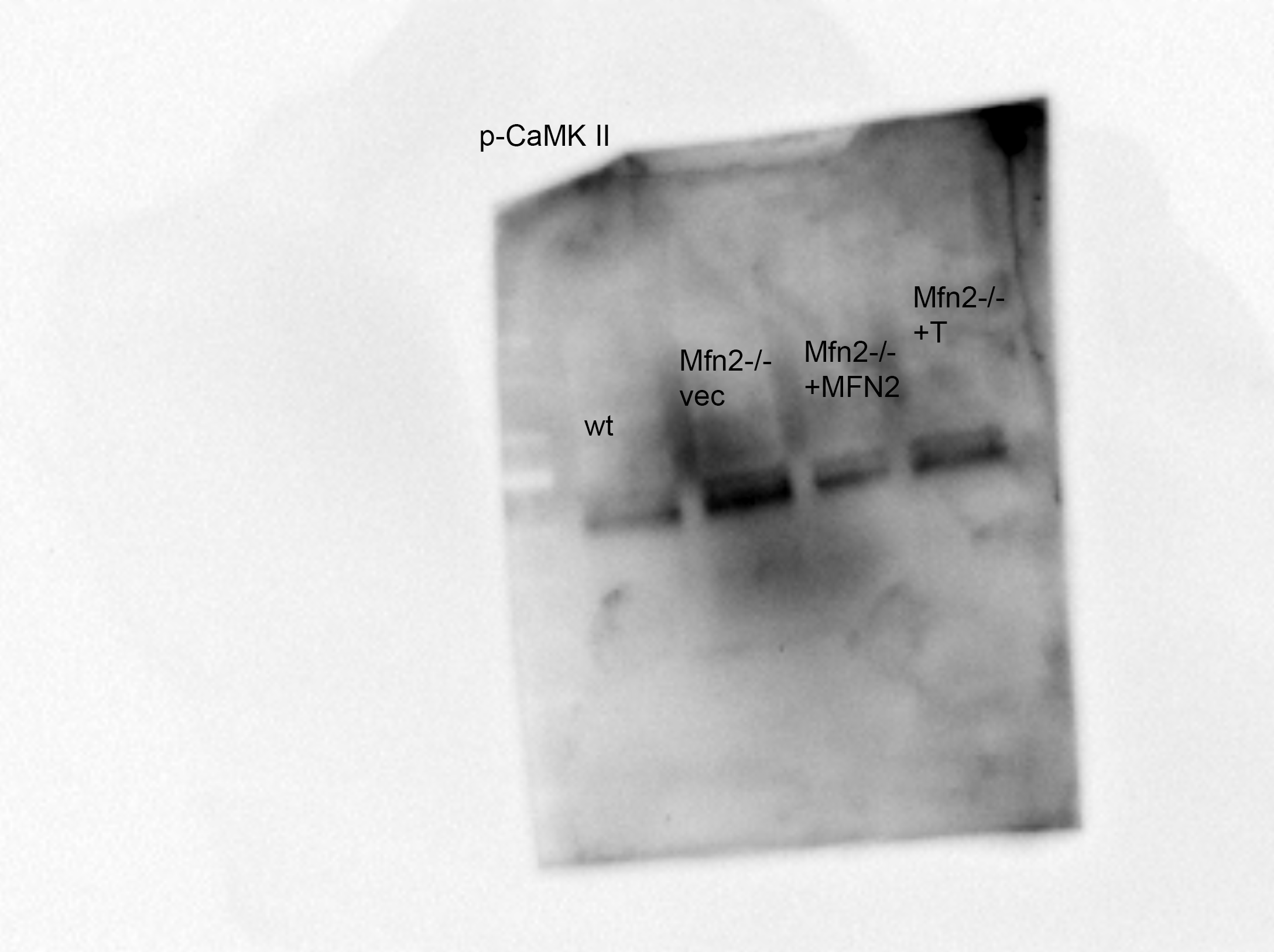

Supplement: Figure 4—source data 1. [file elife-88828-fig4-data1.zip › Figure 4-source data 1/p-CaMKII/Wang, Yueyang 2023-03-02_14h38m07s MEF pCaMK label.tif]

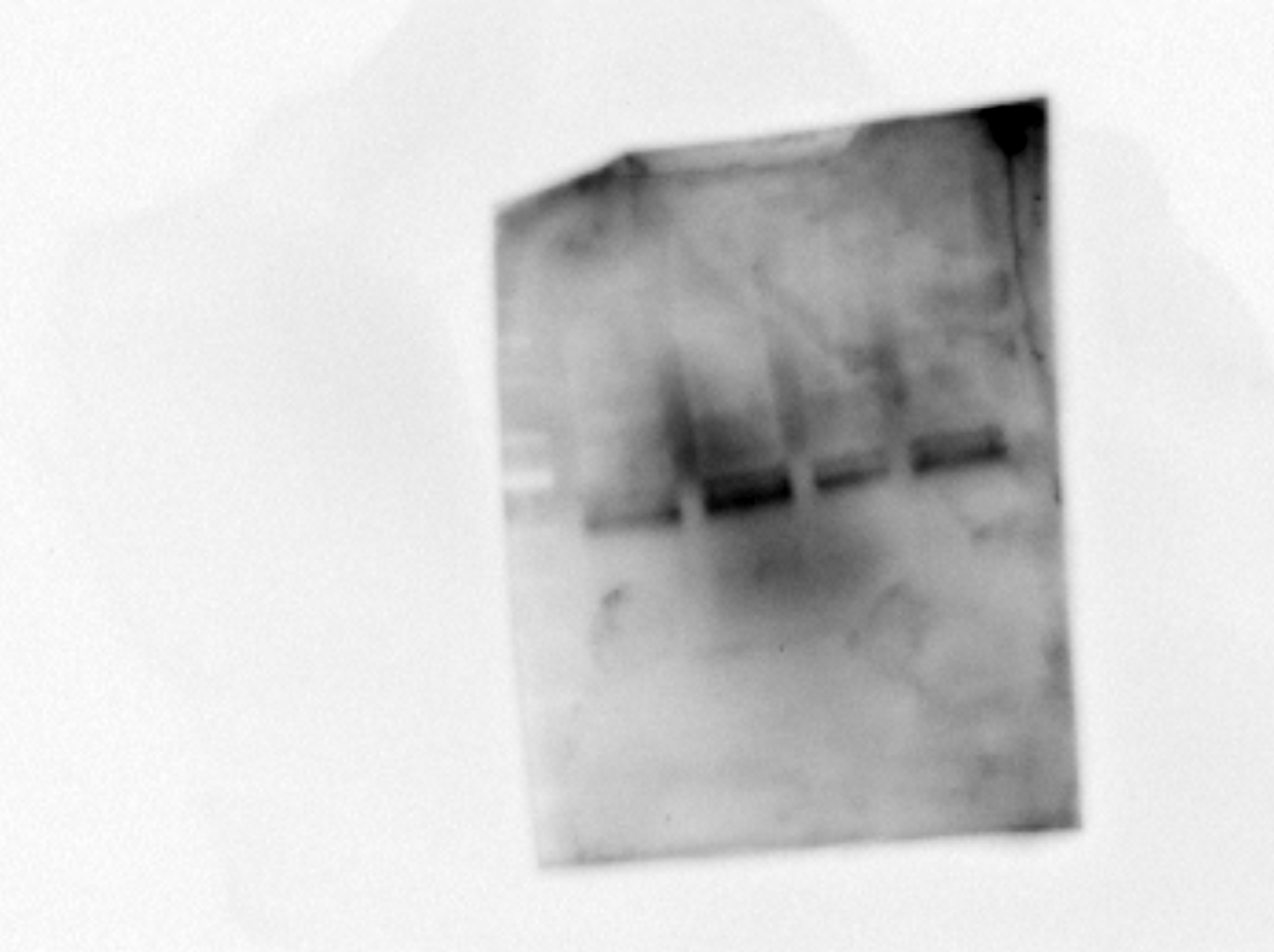

Supplement: Figure 4—source data 1. [file elife-88828-fig4-data1.zip › Figure 4-source data 1/p-CaMKII/Wang, Yueyang 2023-03-02_14h38m07s MEF pCaMK.tif]

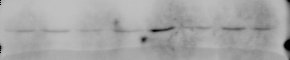

Supplement: Figure 5—source data 1. [file elife-88828-fig5-data1.zip › Figure 5-source data 1/RhoA-GTP/0000651_01_700.jpg]

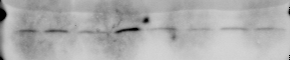

Supplement: Figure 5—source data 1. [file elife-88828-fig5-data1.zip › Figure 5-source data 1/RhoA-GTP/0000651_01_700.TIF-Deuteranope.tif]

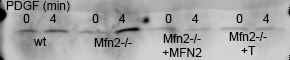

Supplement: Figure 5—source data 1. [file elife-88828-fig5-data1.zip › Figure 5-source data 1/RhoA-GTP/0000651_01_700.TIF-label.tif]

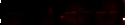

Supplement: Figure 5—source data 1. [file elife-88828-fig5-data1.zip › Figure 5-source data 1/RhoA-GTP/0000651_01_TH.jpg]

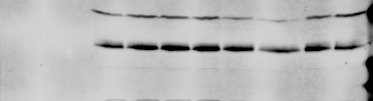

Supplement: Figure 5—source data 1. [file elife-88828-fig5-data1.zip › Figure 5-source data 1/total RhoA/0000649_01_700.jpg]

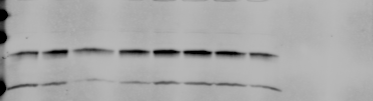

Supplement: Figure 5—source data 1. [file elife-88828-fig5-data1.zip › Figure 5-source data 1/total RhoA/0000649_01_700.TIF-Deuteranope.tif]

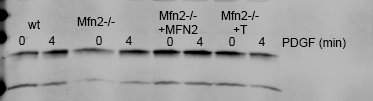

Supplement: Figure 5—source data 1. [file elife-88828-fig5-data1.zip › Figure 5-source data 1/total RhoA/0000649_01_700.TIF-label.tif]

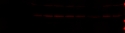

Supplement: Figure 5—source data 1. [file elife-88828-fig5-data1.zip › Figure 5-source data 1/total RhoA/0000649_01_TH.jpg]

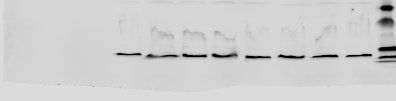

Supplement: Figure 5—source data 1. [file elife-88828-fig5-data1.zip › Figure 5-source data 1/VINCULIn/0000655_01_700.jpg]

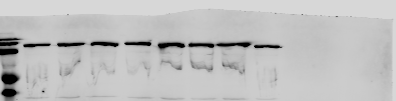

Supplement: Figure 5—source data 1. [file elife-88828-fig5-data1.zip › Figure 5-source data 1/VINCULIn/0000655_01_700.TIF-Deuteranope.tif]

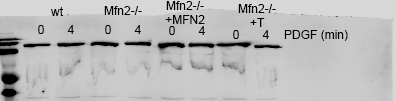

Supplement: Figure 5—source data 1. [file elife-88828-fig5-data1.zip › Figure 5-source data 1/VINCULIn/0000655_01_700.TIF-label.tif]

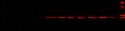

Supplement: Figure 5—source data 1. [file elife-88828-fig5-data1.zip › Figure 5-source data 1/VINCULIn/0000655_01_TH.jpg]

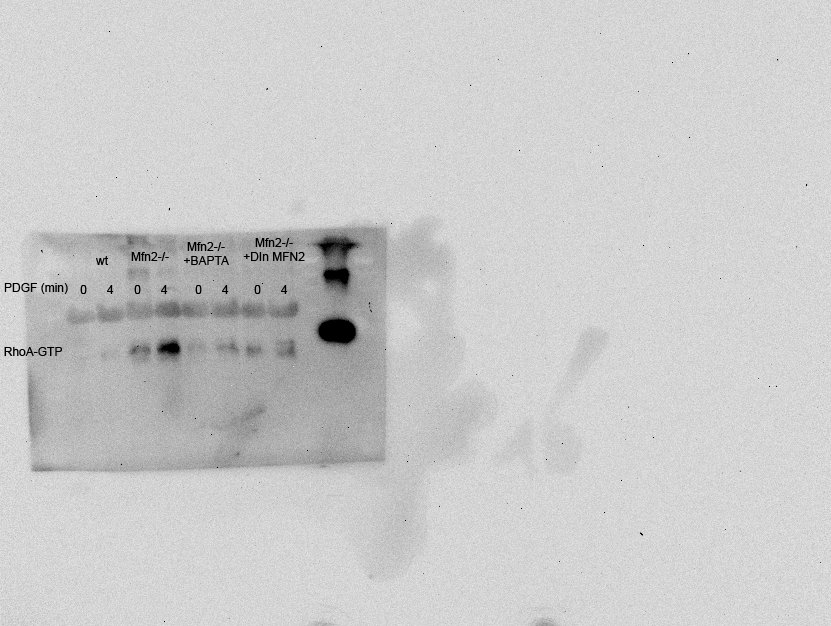

Supplement: Figure 5—source data 2. [file elife-88828-fig5-data2.zip › Figure 5-source data 2/RhoA-GTP/2021-11-04_15-32-04_1_16bit label.tif]

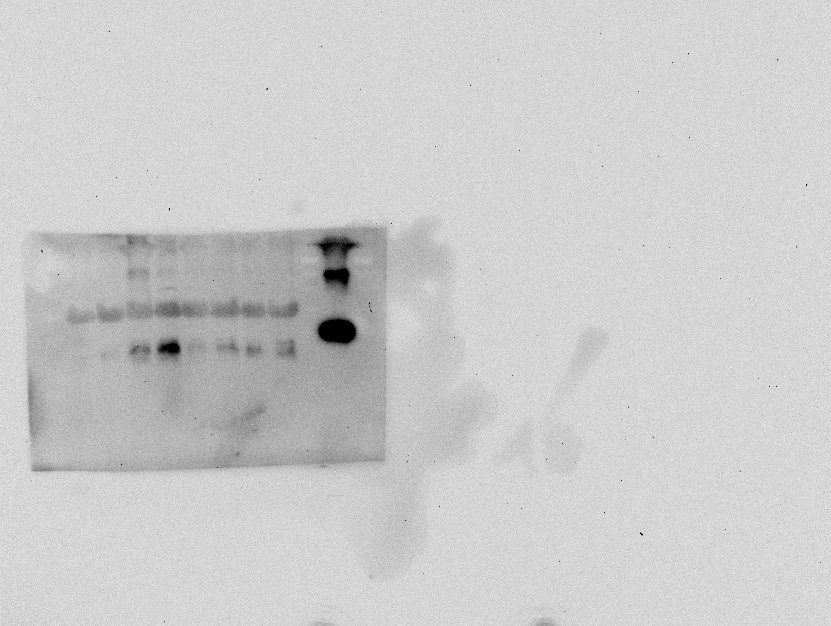

Supplement: Figure 5—source data 2. [file elife-88828-fig5-data2.zip › Figure 5-source data 2/RhoA-GTP/2021-11-04_15-32-04_1_16bit.jpg]

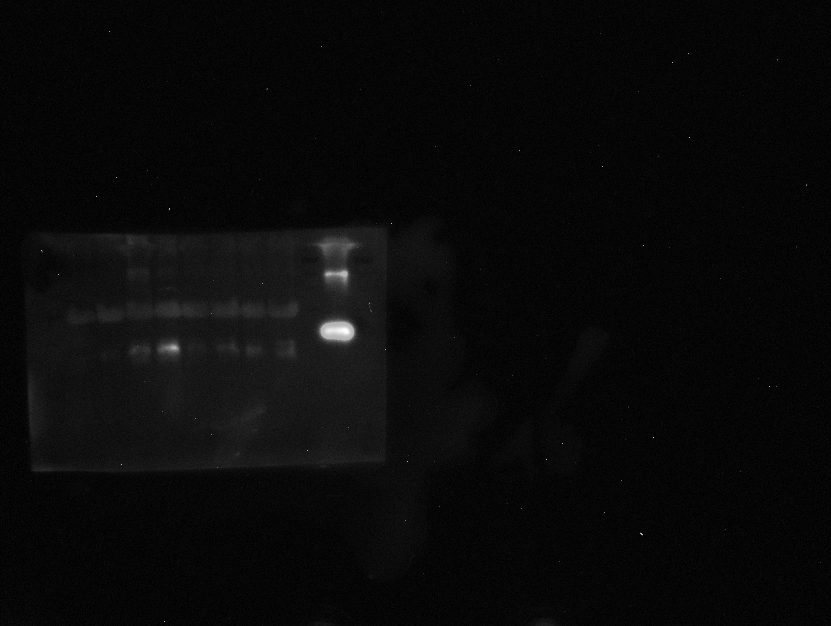

Supplement: Figure 5—source data 2. [file elife-88828-fig5-data2.zip › Figure 5-source data 2/RhoA-GTP/2021-11-04_15-32-04_1_16bit.png]

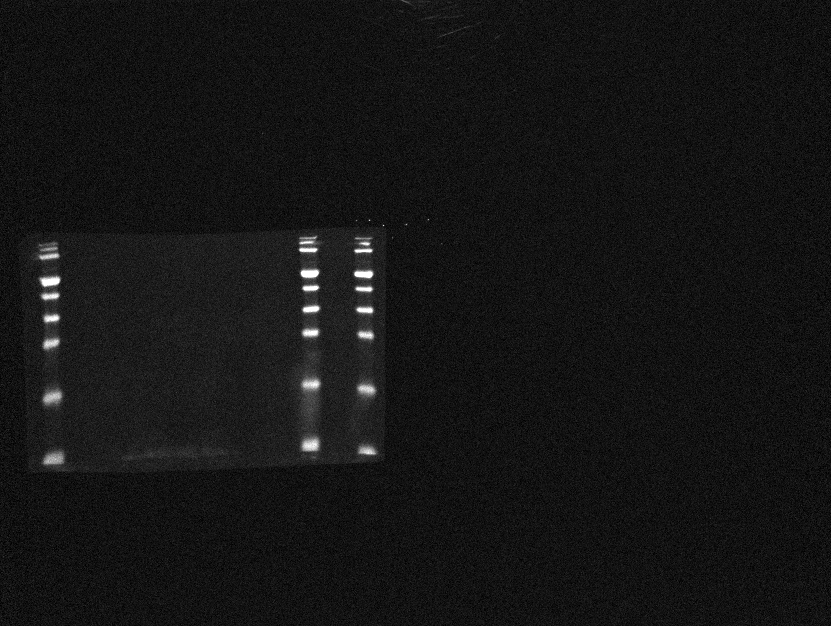

Supplement: Figure 5—source data 2. [file elife-88828-fig5-data2.zip › Figure 5-source data 2/RhoA-GTP/2021-11-04_15-32-04_2_16bit.png]

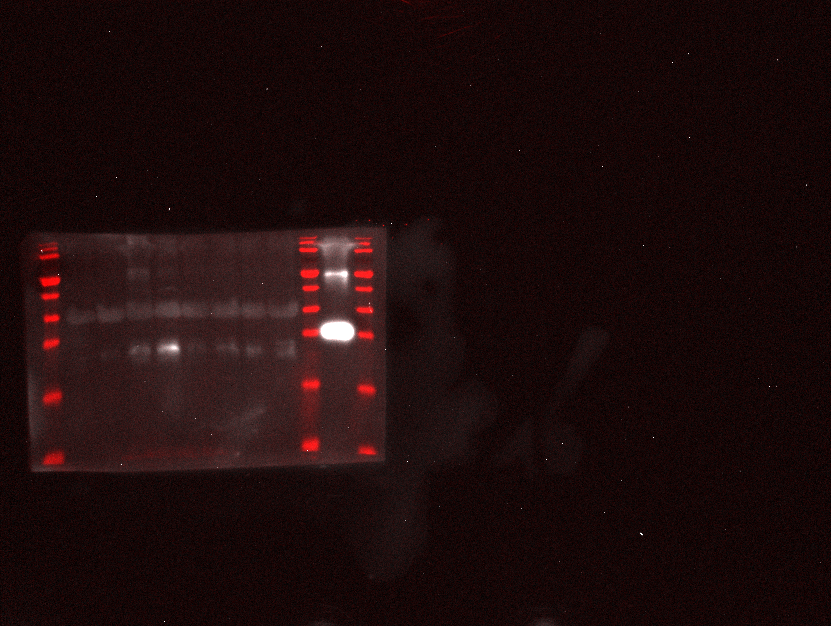

Supplement: Figure 5—source data 2. [file elife-88828-fig5-data2.zip › Figure 5-source data 2/RhoA-GTP/2021-11-04_15-32-04_8bit.png]

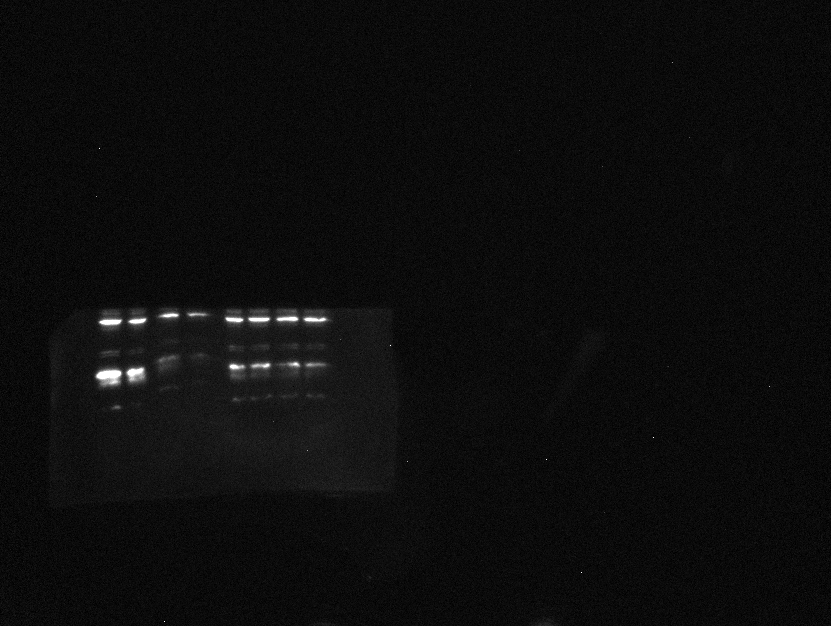

Supplement: Figure 5—source data 2. [file elife-88828-fig5-data2.zip › Figure 5-source data 2/Total RhoA/2021-11-04_15-25-53_1_16bit.png]

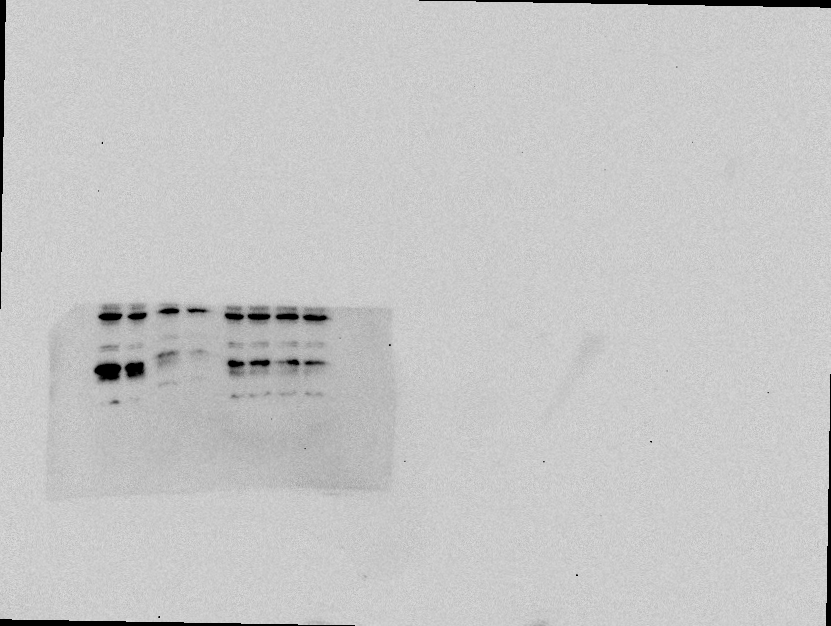

Supplement: Figure 5—source data 2. [file elife-88828-fig5-data2.zip › Figure 5-source data 2/Total RhoA/2021-11-04_15-25-53_1_16bit.png-Deuteranope.tif]

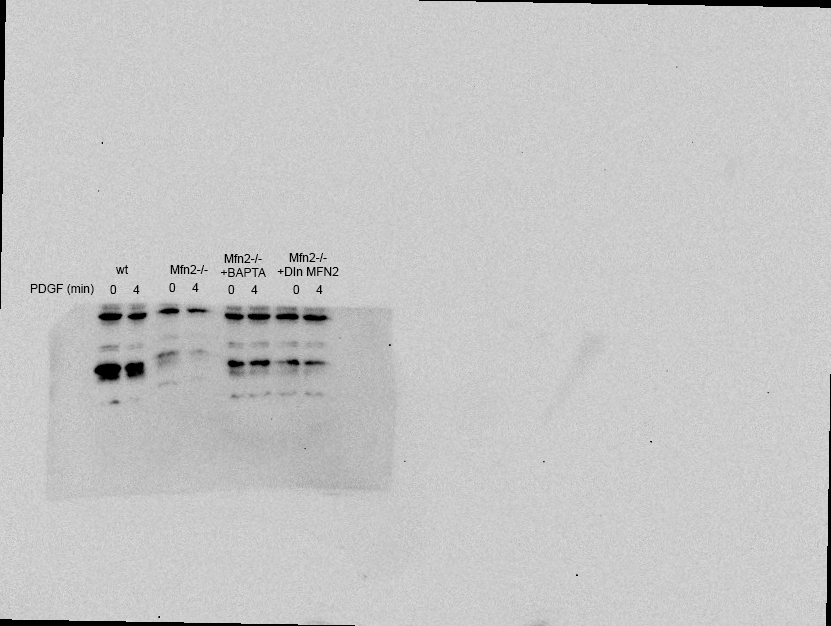

Supplement: Figure 5—source data 2. [file elife-88828-fig5-data2.zip › Figure 5-source data 2/Total RhoA/2021-11-04_15-25-53_1_16bit.png-label.tif]

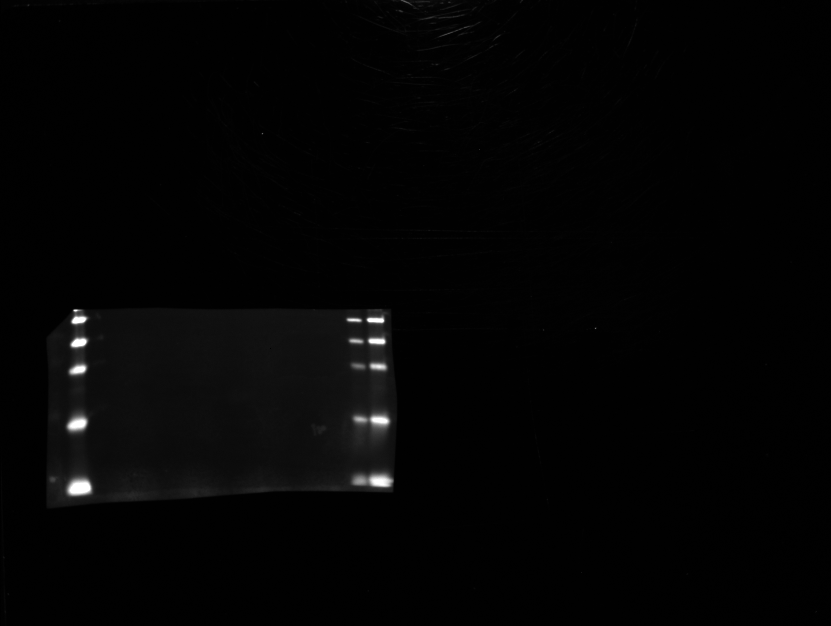

Supplement: Figure 5—source data 2. [file elife-88828-fig5-data2.zip › Figure 5-source data 2/Total RhoA/2021-11-04_15-25-53_2_16bit.png]

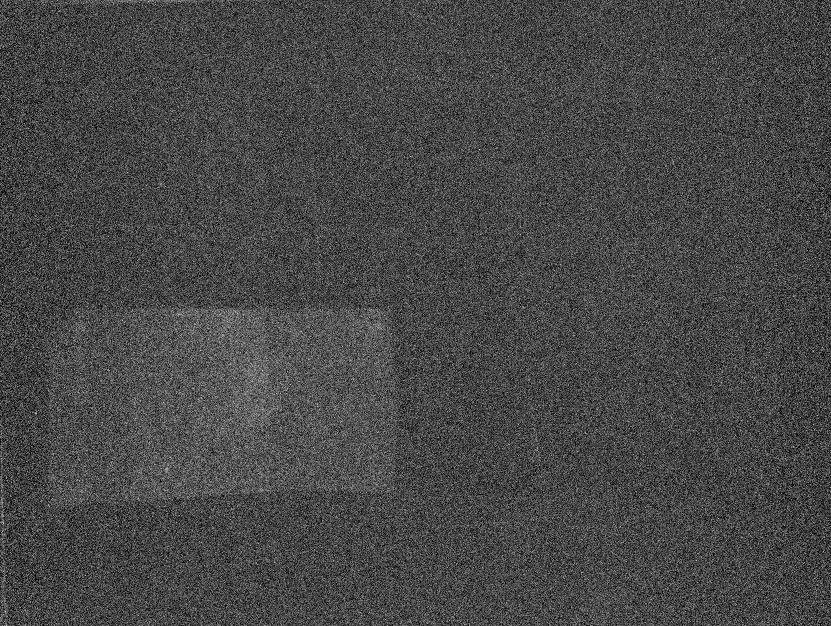

Supplement: Figure 5—source data 2. [file elife-88828-fig5-data2.zip › Figure 5-source data 2/Total RhoA/2021-11-04_15-25-53_3_16bit.png]

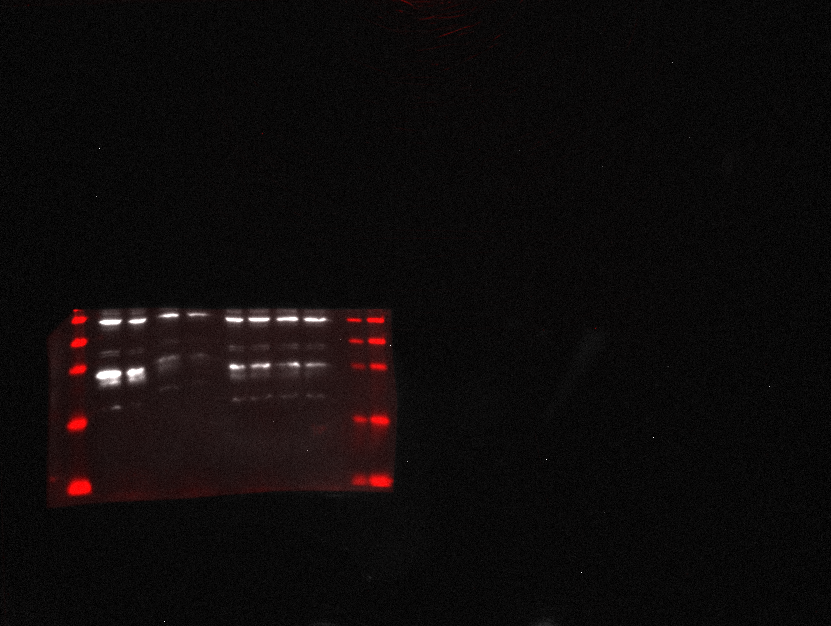

Supplement: Figure 5—source data 2. [file elife-88828-fig5-data2.zip › Figure 5-source data 2/Total RhoA/2021-11-04_15-25-53_8bit.png]

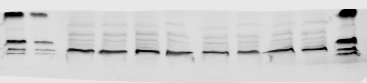

Supplement: Figure 5—source data 2. [file elife-88828-fig5-data2.zip › Figure 5-source data 2/Vinculin/0000697_01_700.jpg]

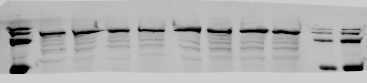

Supplement: Figure 5—source data 2. [file elife-88828-fig5-data2.zip › Figure 5-source data 2/Vinculin/0000697_01_700.TIF-Deuteranope.tif]

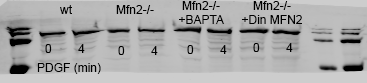

Supplement: Figure 5—source data 2. [file elife-88828-fig5-data2.zip › Figure 5-source data 2/Vinculin/0000697_01_700.TIF-label.tif]

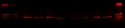

Supplement: Figure 5—source data 2. [file elife-88828-fig5-data2.zip › Figure 5-source data 2/Vinculin/0000697_01_TH.jpg]

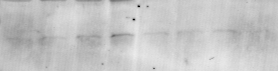

Supplement: Figure 5—figure supplement 1—source data 1. [file elife-88828-fig5-figsupp1-data1.zip › Figure 5-figure supplement 1-source data 1/Rac-GTP/0000638_01_700 2.tif-Deuteranope.tif]

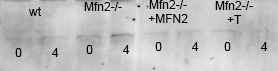

Supplement: Figure 5—figure supplement 1—source data 1. [file elife-88828-fig5-figsupp1-data1.zip › Figure 5-figure supplement 1-source data 1/Rac-GTP/0000638_01_700 2.tif-label.tif]

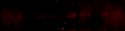

Supplement: Figure 5—figure supplement 1—source data 1. [file elife-88828-fig5-figsupp1-data1.zip › Figure 5-figure supplement 1-source data 1/Rac-GTP/0000638_01_TH.jpg]

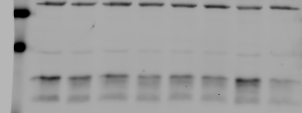

Supplement: Figure 5—figure supplement 1—source data 1. [file elife-88828-fig5-figsupp1-data1.zip › Figure 5-figure supplement 1-source data 1/Total RAC/0000641_01_700.TIF-Deuteranope.tif]

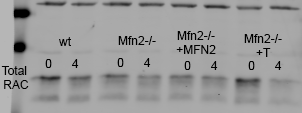

Supplement: Figure 5—figure supplement 1—source data 1. [file elife-88828-fig5-figsupp1-data1.zip › Figure 5-figure supplement 1-source data 1/Total RAC/0000641_01_700.TIF-label.tif]

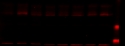

Supplement: Figure 5—figure supplement 1—source data 1. [file elife-88828-fig5-figsupp1-data1.zip › Figure 5-figure supplement 1-source data 1/Total RAC/0000641_01_TH.jpg]

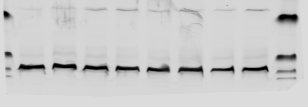

Supplement: Figure 5—figure supplement 1—source data 1. [file elife-88828-fig5-figsupp1-data1.zip › Figure 5-figure supplement 1-source data 1/Vinculin/0000646_01_700.jpg]

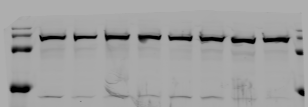

Supplement: Figure 5—figure supplement 1—source data 1. [file elife-88828-fig5-figsupp1-data1.zip › Figure 5-figure supplement 1-source data 1/Vinculin/0000646_01_700.TIF-Deuteranope.tif]

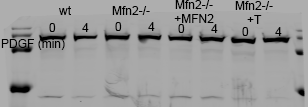

Supplement: Figure 5—figure supplement 1—source data 1. [file elife-88828-fig5-figsupp1-data1.zip › Figure 5-figure supplement 1-source data 1/Vinculin/0000646_01_700.TIF-label.tif]

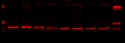

Supplement: Figure 5—figure supplement 1—source data 1. [file elife-88828-fig5-figsupp1-data1.zip › Figure 5-figure supplement 1-source data 1/Vinculin/0000646_01_TH.jpg]

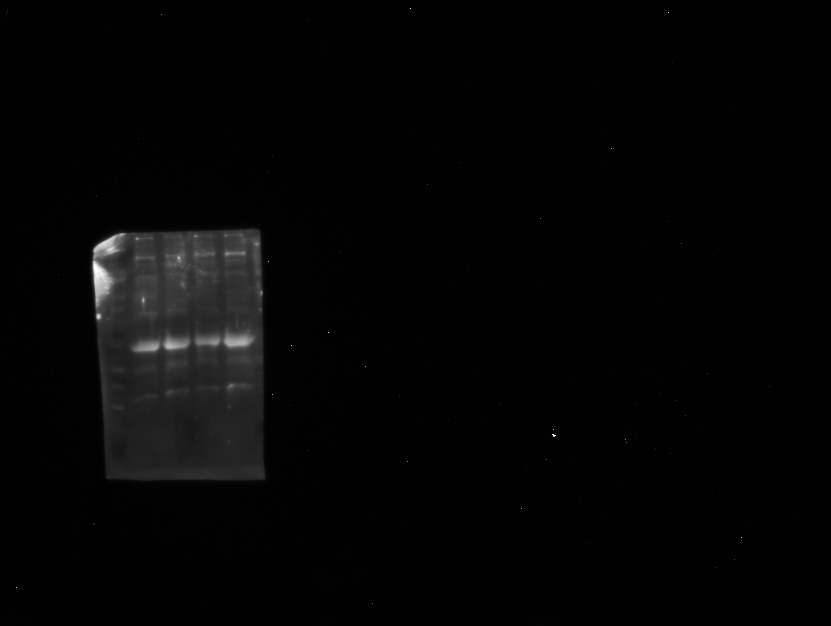

Supplement: Figure 5—figure supplement 1—source data 2. [file elife-88828-fig5-figsupp1-data2.zip › Figure 5-figure supplement 1-source data 2/CDC42-GTP/2022-05-19_09-11-56 Cdc42_1_16bit.png]

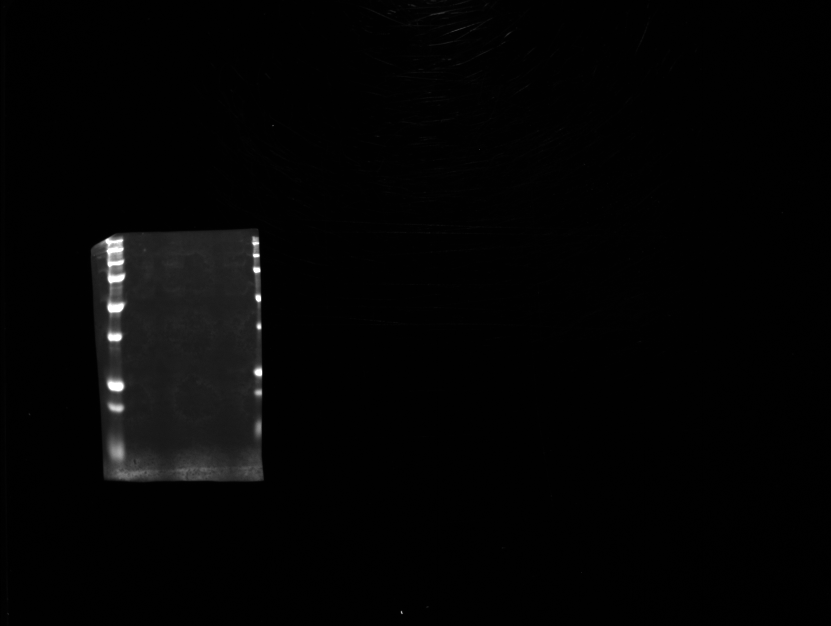

Supplement: Figure 5—figure supplement 1—source data 2. [file elife-88828-fig5-figsupp1-data2.zip › Figure 5-figure supplement 1-source data 2/CDC42-GTP/2022-05-19_09-11-56 Cdc42_2_16bit.png]

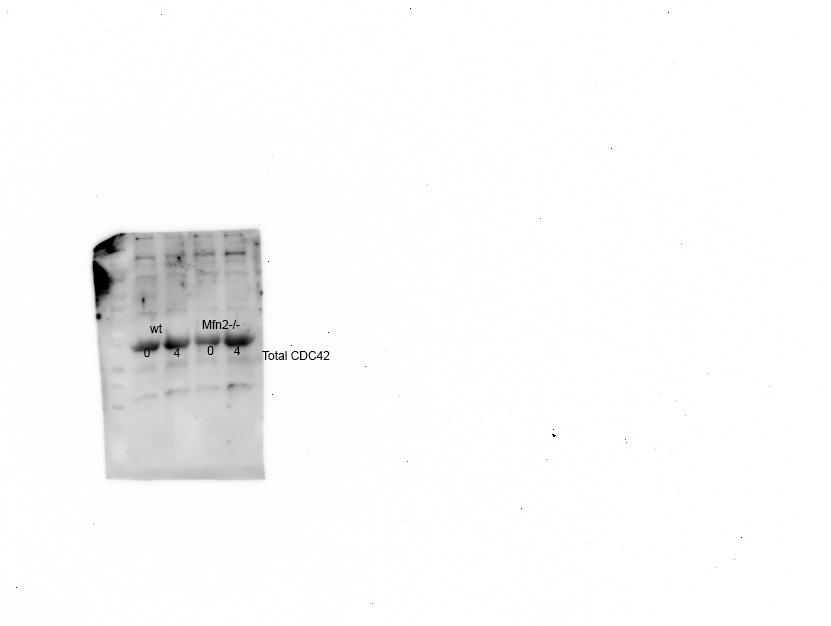

Supplement: Figure 5—figure supplement 1—source data 2. [file elife-88828-fig5-figsupp1-data2.zip › Figure 5-figure supplement 1-source data 2/CDC42-GTP/2022-05-19_09-11-56 Cdc42_8bit label.tif]

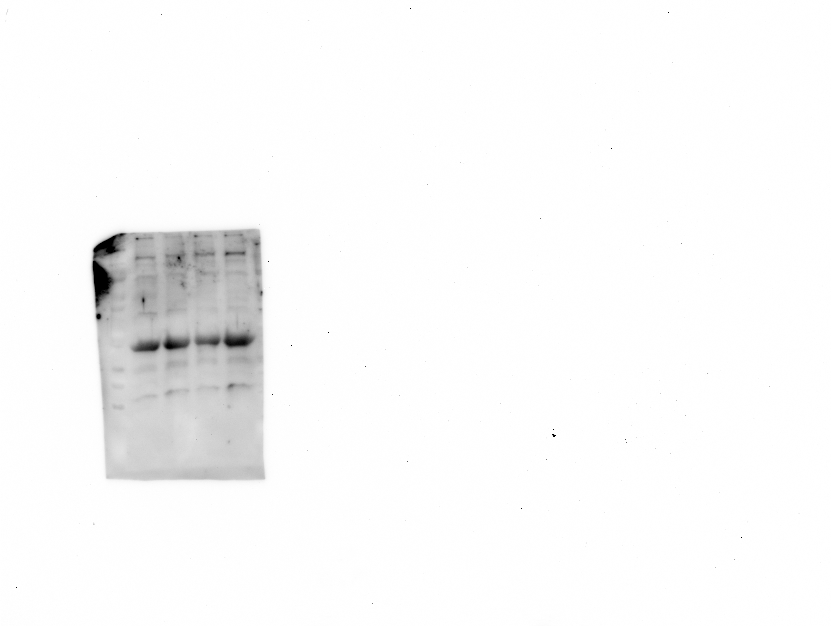

Supplement: Figure 5—figure supplement 1—source data 2. [file elife-88828-fig5-figsupp1-data2.zip › Figure 5-figure supplement 1-source data 2/CDC42-GTP/2022-05-19_09-11-56 Cdc42_8bit.png]

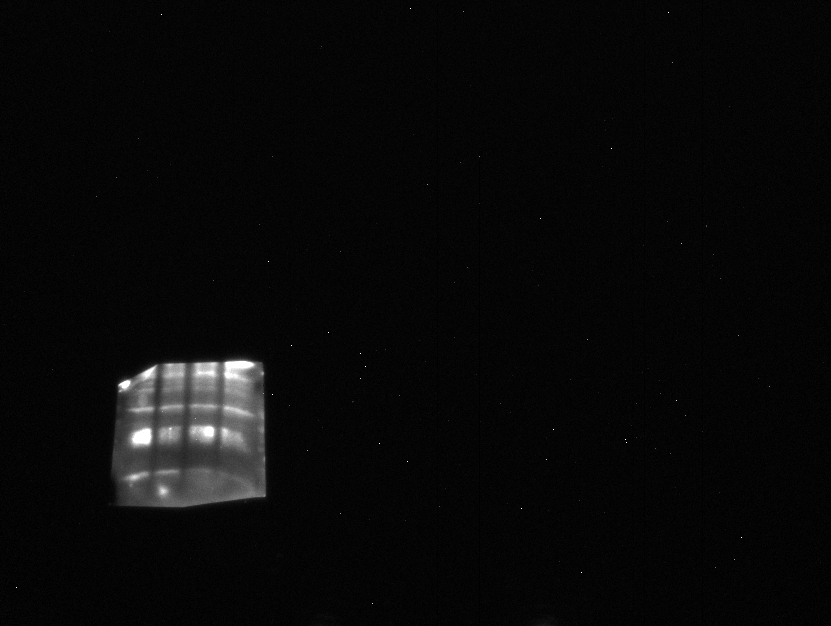

Supplement: Figure 5—figure supplement 1—source data 2. [file elife-88828-fig5-figsupp1-data2.zip › Figure 5-figure supplement 1-source data 2/Total CDC42/2022-05-19_09-36-59 Cdc42 total_1_16bit.png]

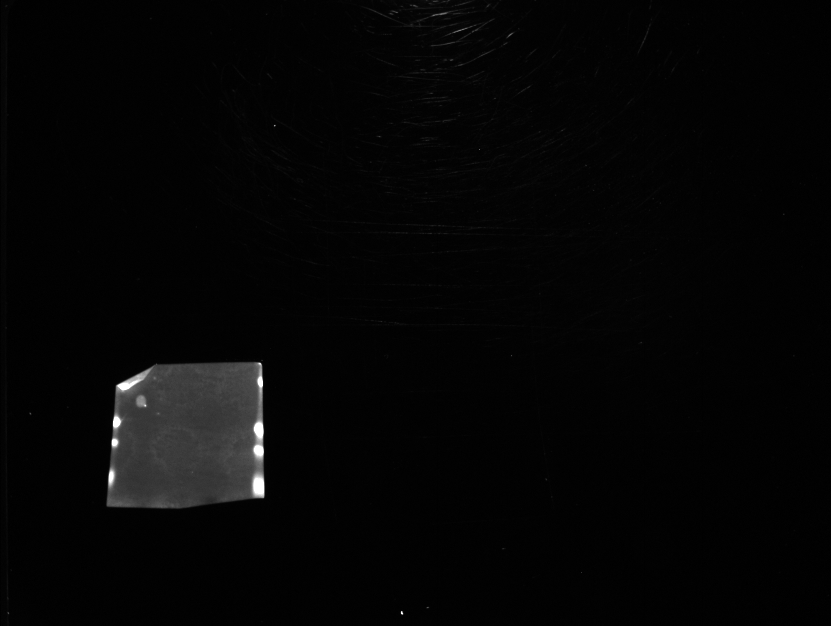

Supplement: Figure 5—figure supplement 1—source data 2. [file elife-88828-fig5-figsupp1-data2.zip › Figure 5-figure supplement 1-source data 2/Total CDC42/2022-05-19_09-36-59 Cdc42 total_2_16bit.png]

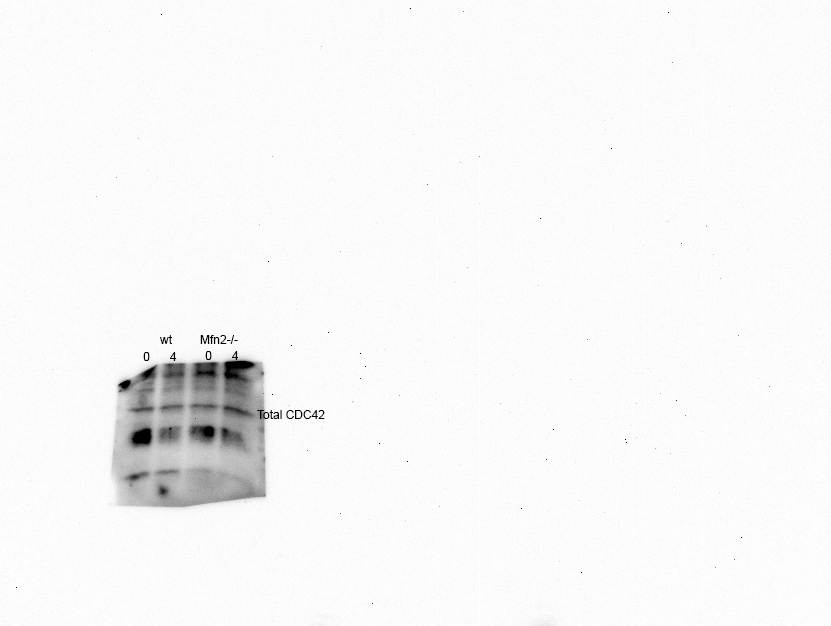

Supplement: Figure 5—figure supplement 1—source data 2. [file elife-88828-fig5-figsupp1-data2.zip › Figure 5-figure supplement 1-source data 2/Total CDC42/2022-05-19_09-36-59 Cdc42 total_8bit label.tif]

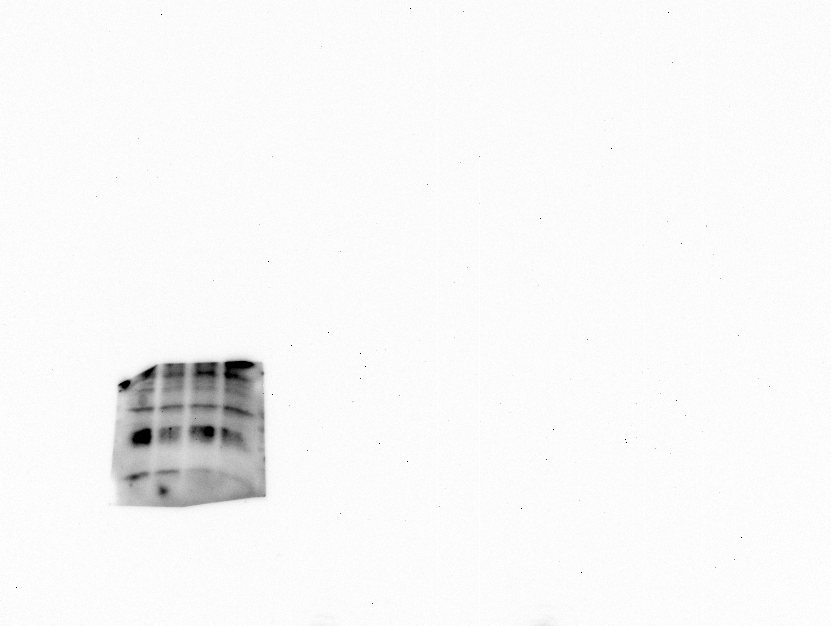

Supplement: Figure 5—figure supplement 1—source data 2. [file elife-88828-fig5-figsupp1-data2.zip › Figure 5-figure supplement 1-source data 2/Total CDC42/2022-05-19_09-36-59 Cdc42 total_8bit.png]

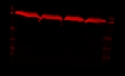

Supplement: Figure 5—figure supplement 1—source data 2. [file elife-88828-fig5-figsupp1-data2.zip › Figure 5-figure supplement 1-source data 2/TUBULIN/0000770_01/0000770_01_TH.jpg]

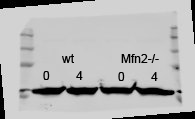

Supplement: Figure 5—figure supplement 1—source data 2. [file elife-88828-fig5-figsupp1-data2.zip › Figure 5-figure supplement 1-source data 2/TUBULIN/0515 pulldown tubulin label.tif]

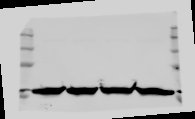

Supplement: Figure 5—figure supplement 1—source data 2. [file elife-88828-fig5-figsupp1-data2.zip › Figure 5-figure supplement 1-source data 2/TUBULIN/0515 pulldown tubulin.jpg]

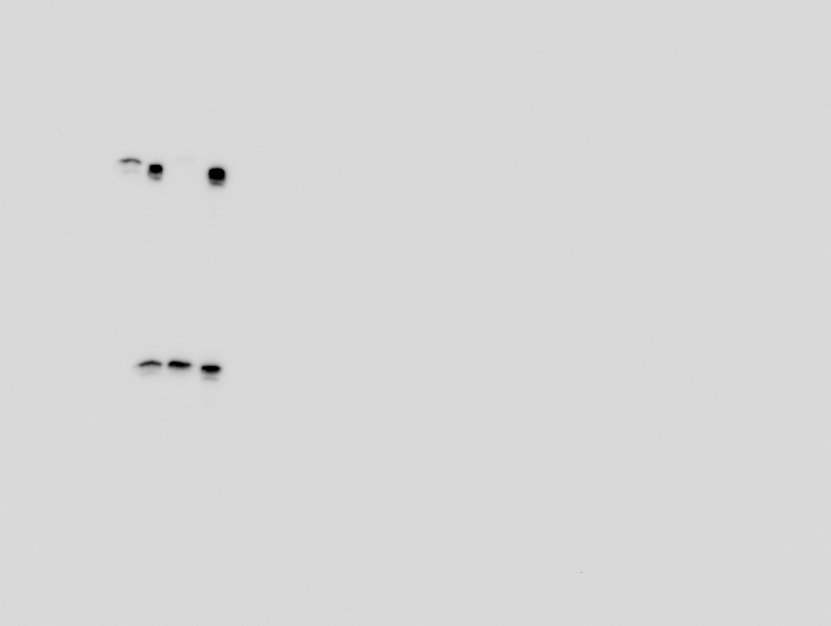

Supplement: Figure 7—source data 1. [file elife-88828-fig7-data1.zip › Figure 7-source data 1/MLC II/Mlc_1_16bit.jpg]

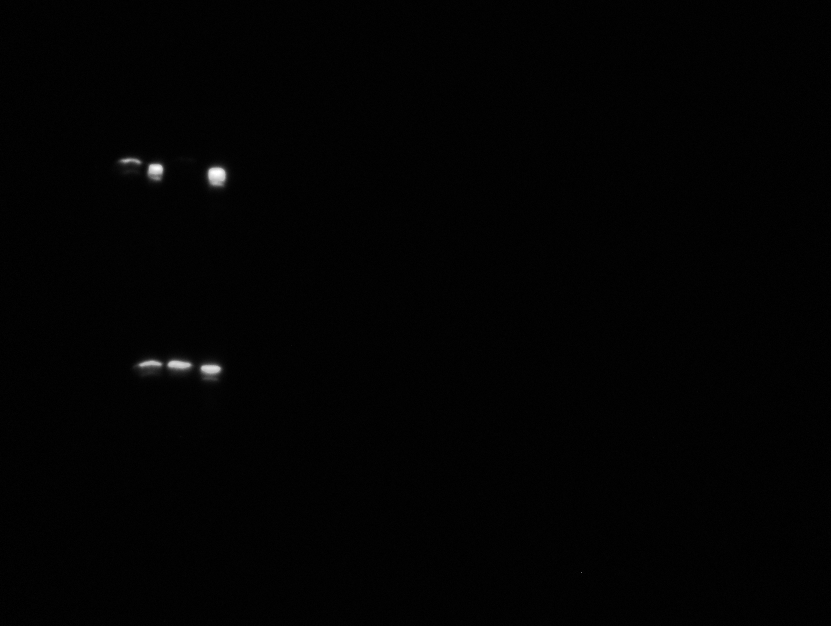

Supplement: Figure 7—source data 1. [file elife-88828-fig7-data1.zip › Figure 7-source data 1/MLC II/Mlc_1_16bit.png]

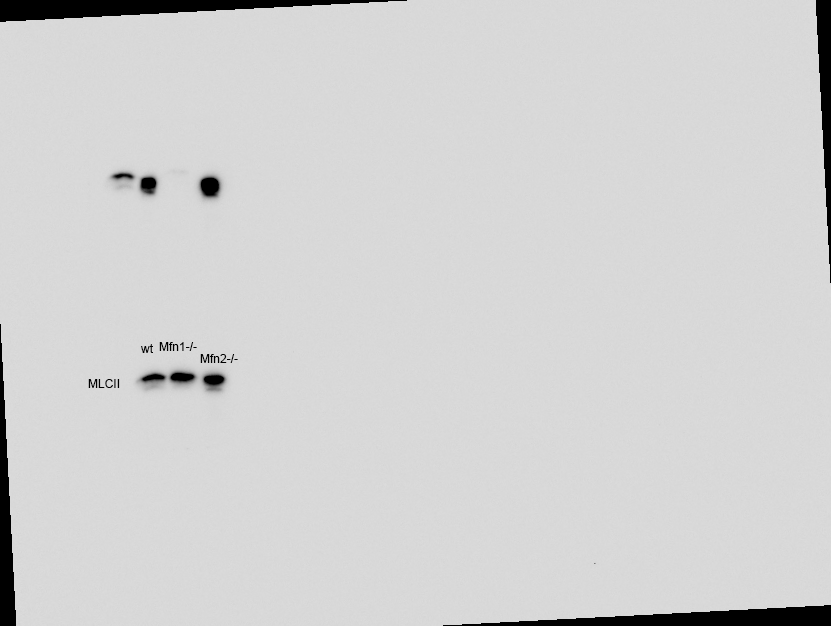

Supplement: Figure 7—source data 1. [file elife-88828-fig7-data1.zip › Figure 7-source data 1/MLC II/Mlc_1_16bit.png-label.tif]

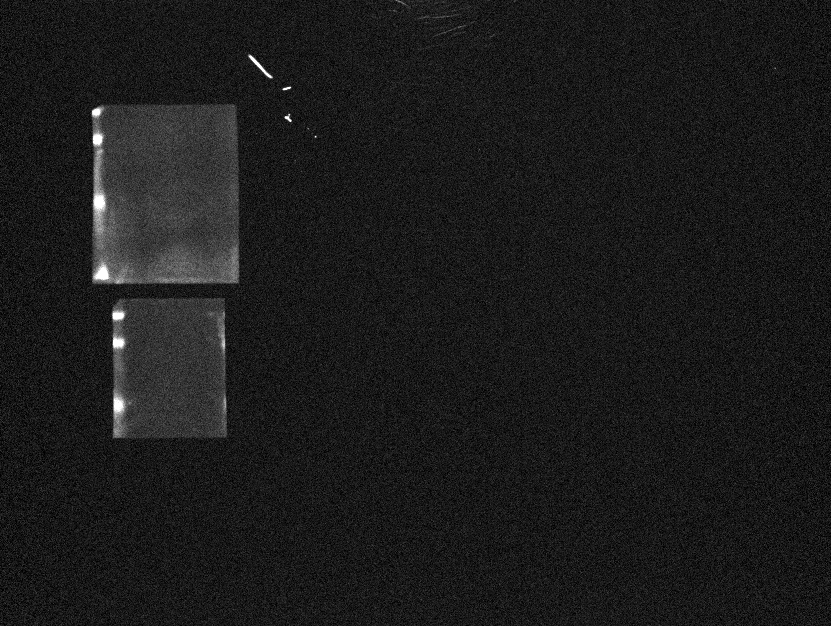

Supplement: Figure 7—source data 1. [file elife-88828-fig7-data1.zip › Figure 7-source data 1/MLC II/Mlc_2_16bit.png]

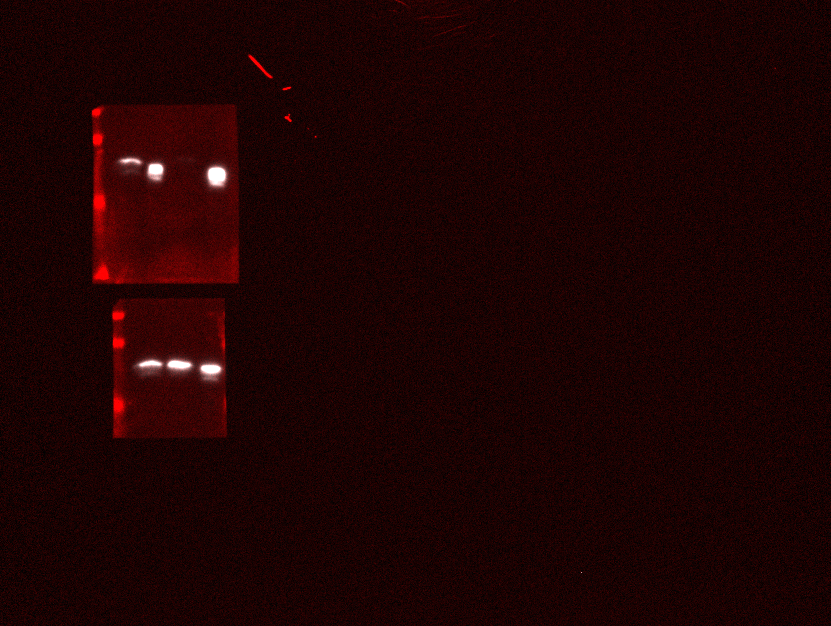

Supplement: Figure 7—source data 1. [file elife-88828-fig7-data1.zip › Figure 7-source data 1/MLC II/Mlc_8bit.png]

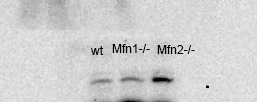

Supplement: Figure 7—source data 1. [file elife-88828-fig7-data1.zip › Figure 7-source data 1/pMLC II/2021-11-23_12-30-26_1_16bit.png-label.tif]

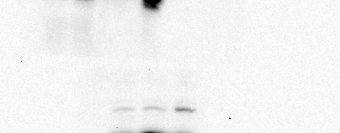

Supplement: Figure 7—source data 1. [file elife-88828-fig7-data1.zip › Figure 7-source data 1/pMLC II/2021-11-23_12-30-26_1_16bit.tif]

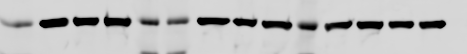

Supplement: Figure 7—source data 1. [file elife-88828-fig7-data1.zip › Figure 7-source data 1/Tubulin/0000740_01_700.TIF-Deuteranope.tif]

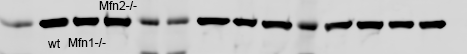

Supplement: Figure 7—source data 1. [file elife-88828-fig7-data1.zip › Figure 7-source data 1/Tubulin/0000740_01_700.TIF-label.tif]

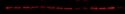

Supplement: Figure 7—source data 1. [file elife-88828-fig7-data1.zip › Figure 7-source data 1/Tubulin/0000740_01_TH.jpg]

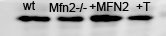

Supplement: Figure 7—source data 2. [file elife-88828-fig7-data2.zip › Figure 7-source data 2/MLCII/0000753_01_800 2 label.tif]

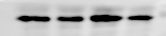

Supplement: Figure 7—source data 2. [file elife-88828-fig7-data2.zip › Figure 7-source data 2/MLCII/0000753_01_800 2.tif]

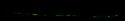

Supplement: Figure 7—source data 2. [file elife-88828-fig7-data2.zip › Figure 7-source data 2/MLCII/0000753_01_TH.jpg]

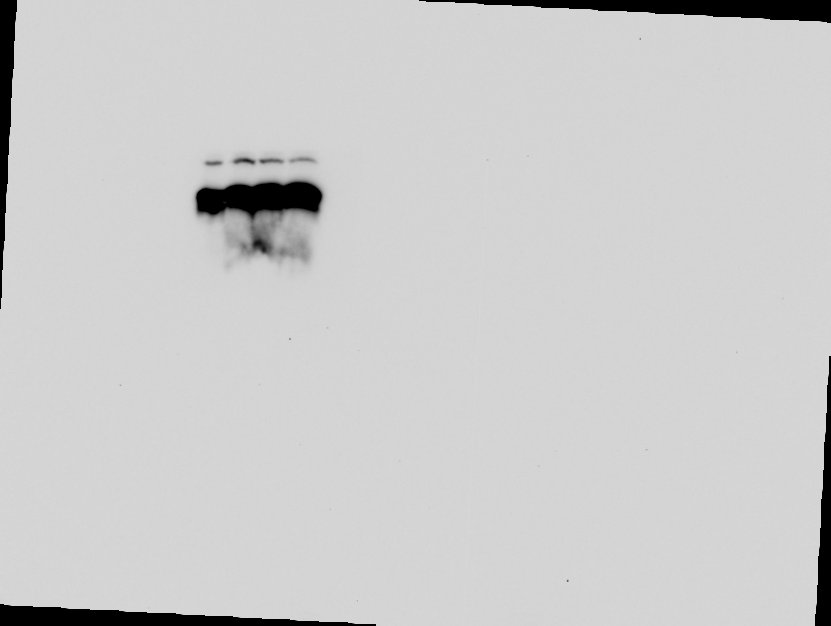

Supplement: Figure 7—source data 2. [file elife-88828-fig7-data2.zip › Figure 7-source data 2/pMLCII/2021-11-28_14-34-34 Pmlc2_1_16bit.jpg]

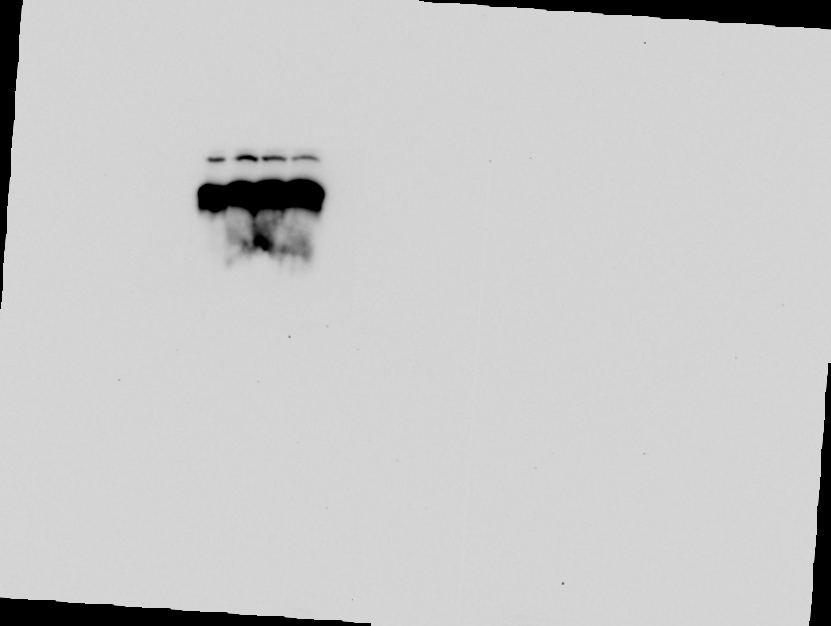

Supplement: Figure 7—source data 2. [file elife-88828-fig7-data2.zip › Figure 7-source data 2/pMLCII/2021-11-28_14-34-34 Pmlc2_1_16bit.png-Deuteranope.tif]

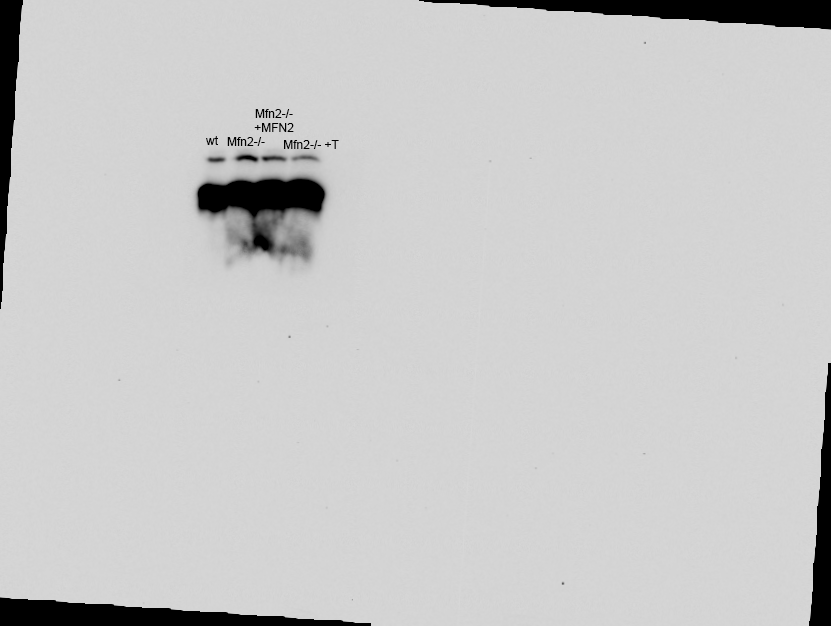

Supplement: Figure 7—source data 2. [file elife-88828-fig7-data2.zip › Figure 7-source data 2/pMLCII/2021-11-28_14-34-34 Pmlc2_1_16bit.png-label.tif]

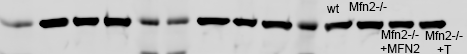

Supplement: Figure 7—source data 2. [file elife-88828-fig7-data2.zip › Figure 7-source data 2/TUBULIN/0000740_01_700.TIF-Label.tif]

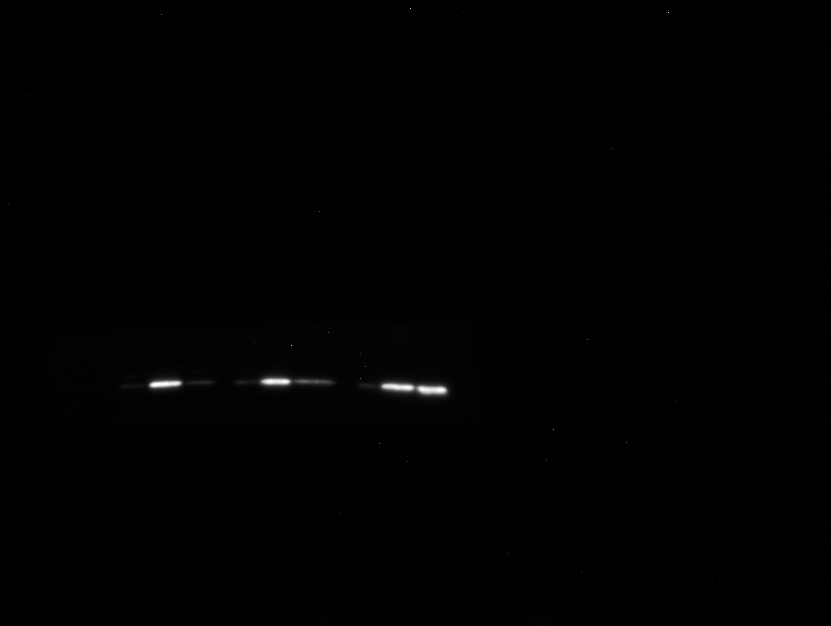

Supplement: Figure 7—source data 3. [file elife-88828-fig7-data3.zip › Figure 7-source data 3/pMLCII/2022-02-16_14-09-57 Gel3 pmlc_1_16bit.png]

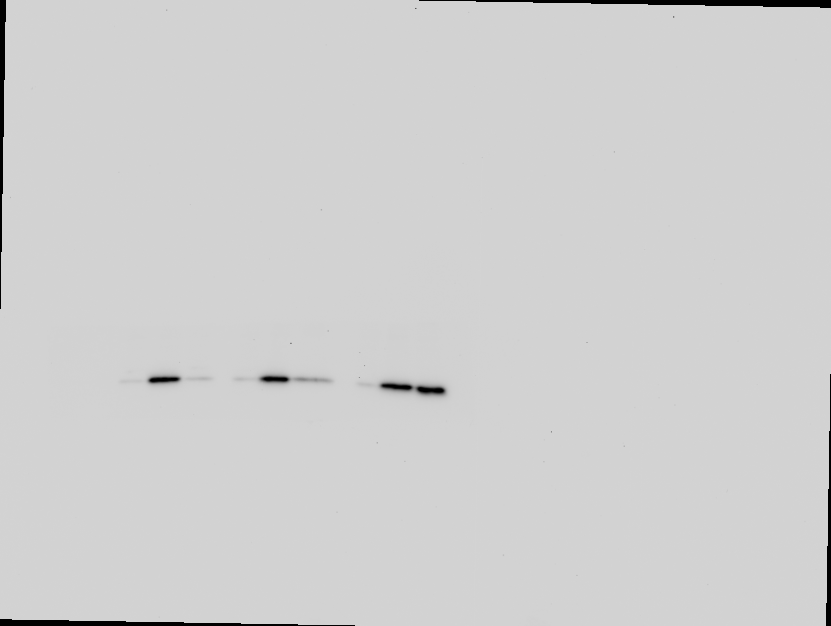

Supplement: Figure 7—source data 3. [file elife-88828-fig7-data3.zip › Figure 7-source data 3/pMLCII/2022-02-16_14-09-57 Gel3 pmlc_1_16bit.png-Deuteranope 2.tif]

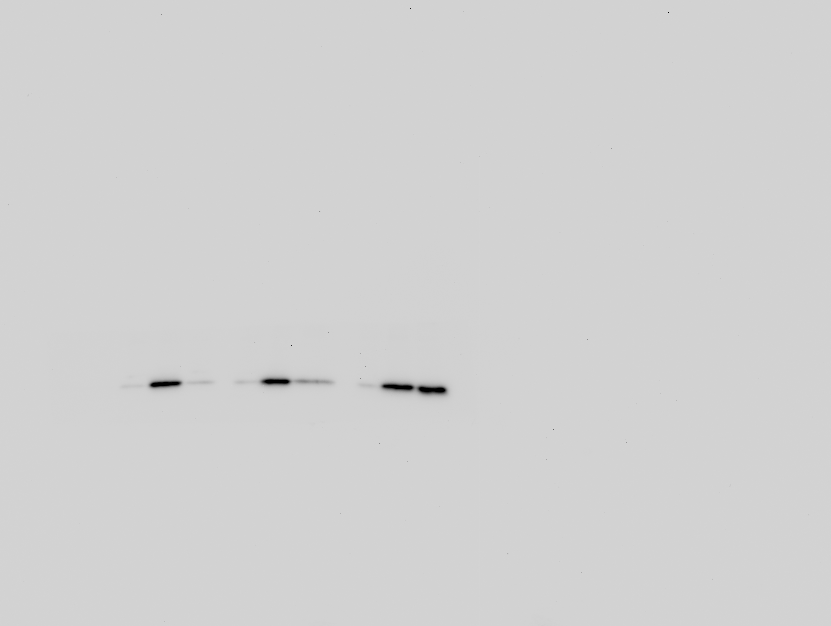

Supplement: Figure 7—source data 3. [file elife-88828-fig7-data3.zip › Figure 7-source data 3/pMLCII/2022-02-16_14-09-57 Gel3 pmlc_1_16bit.png-Deuteranope.tif]

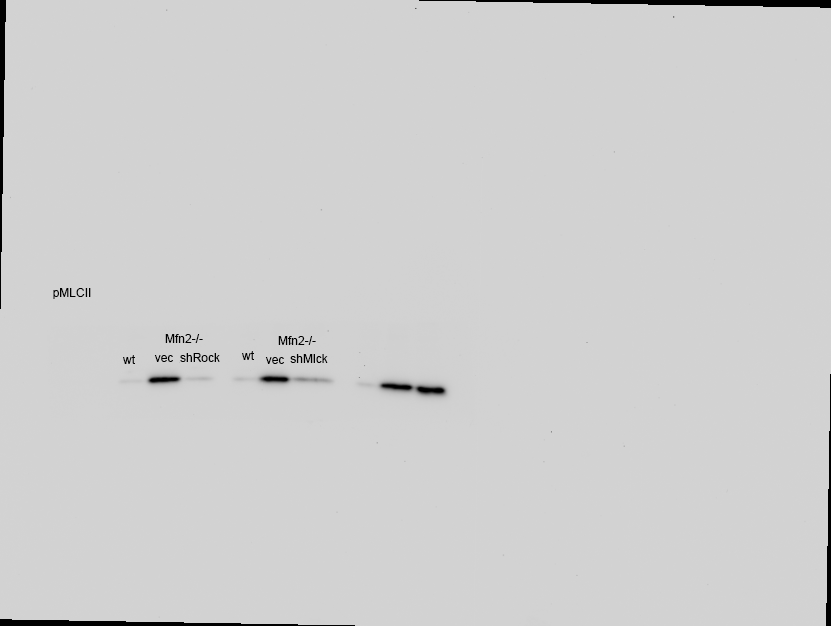

Supplement: Figure 7—source data 3. [file elife-88828-fig7-data3.zip › Figure 7-source data 3/pMLCII/2022-02-16_14-09-57 Gel3 pmlc_1_16bit.png-label.tif]

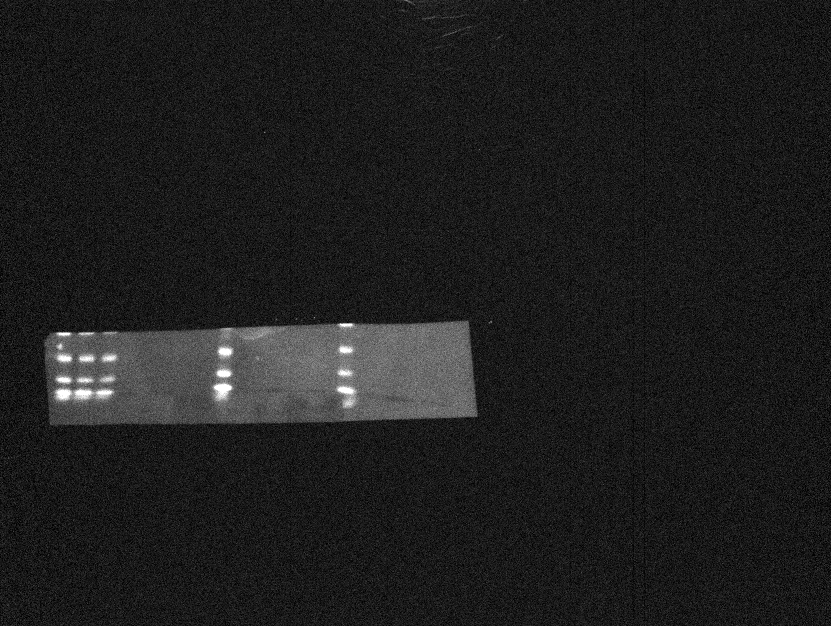

Supplement: Figure 7—source data 3. [file elife-88828-fig7-data3.zip › Figure 7-source data 3/pMLCII/2022-02-16_14-09-57 Gel3 pmlc_2_16bit.png]

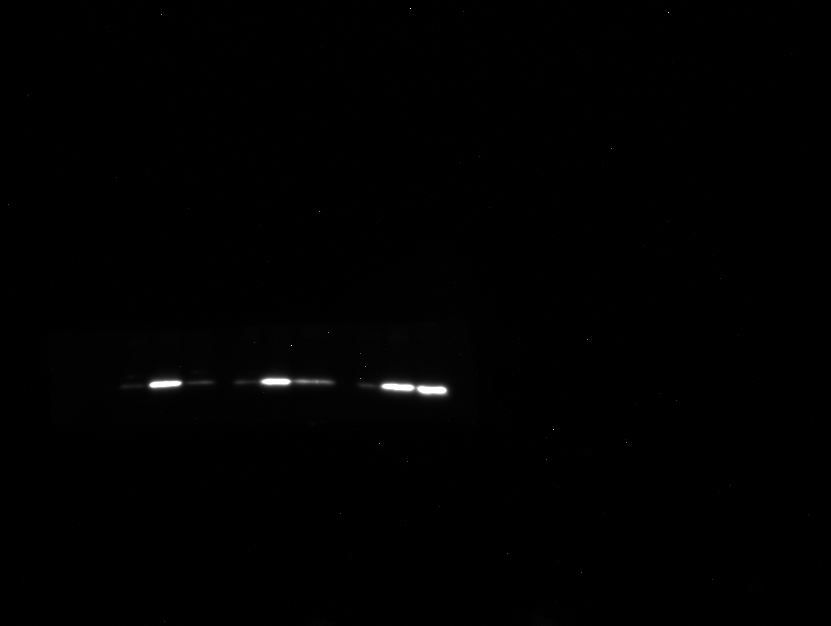

Supplement: Figure 7—source data 3. [file elife-88828-fig7-data3.zip › Figure 7-source data 3/pMLCII/2022-02-16_14-09-57 Gel3 pmlc_8bit.png]

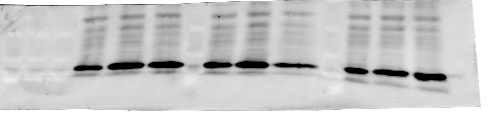

Supplement: Figure 7—source data 3. [file elife-88828-fig7-data3.zip › Figure 7-source data 3/Total MLCII/0000749_01_800.TIF-Deuteranope.tif]

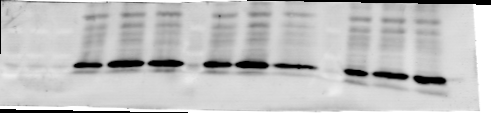

Supplement: Figure 7—source data 3. [file elife-88828-fig7-data3.zip › Figure 7-source data 3/Total MLCII/0000749_01_800.TIF-Deuteranope2.tif]

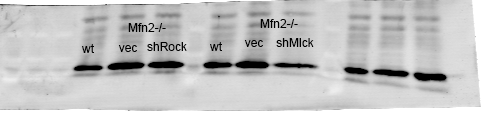

Supplement: Figure 7—source data 3. [file elife-88828-fig7-data3.zip › Figure 7-source data 3/Total MLCII/0000749_01_800.TIF-label.tif]

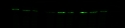

Supplement: Figure 7—source data 3. [file elife-88828-fig7-data3.zip › Figure 7-source data 3/Total MLCII/0000749_01_TH.jpg]

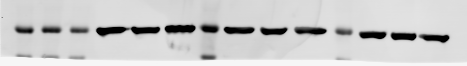

Supplement: Figure 7—source data 3. [file elife-88828-fig7-data3.zip › Figure 7-source data 3/TUBULIN/0000742_01_700.TIF-Deuteranope.tif]

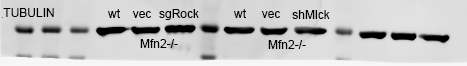

Supplement: Figure 7—source data 3. [file elife-88828-fig7-data3.zip › Figure 7-source data 3/TUBULIN/0000742_01_700.TIF-label.tif]

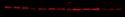

Supplement: Figure 7—source data 3. [file elife-88828-fig7-data3.zip › Figure 7-source data 3/TUBULIN/0000742_01_TH.jpg]

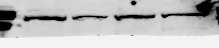

Supplement: Figure 7—source data 4. [file elife-88828-fig7-data4.zip › Figure 7-source data 4/MLCK/0000728_01_700.TIF-Deuteranope.tif]

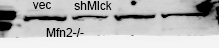

Supplement: Figure 7—source data 4. [file elife-88828-fig7-data4.zip › Figure 7-source data 4/MLCK/0000728_01_700.TIF-label.tif]
