# Supplementary figures and images for: Atypical peripheral actin band formation via overactivation of RhoA and nonmuscle myosin II in mitofusin 2-deficient cells (part 2 of 2)
Source: eLife. 2023 Sep 19;12:e88828. doi: 10.7554/eLife.88828 (PMC10550287; doi:10.7554/eLife.88828)

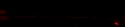

Supplement: Figure 7—source data 4. [file elife-88828-fig7-data4.zip › Figure 7-source data 4/MLCK/0000728_01_TH.jpg]

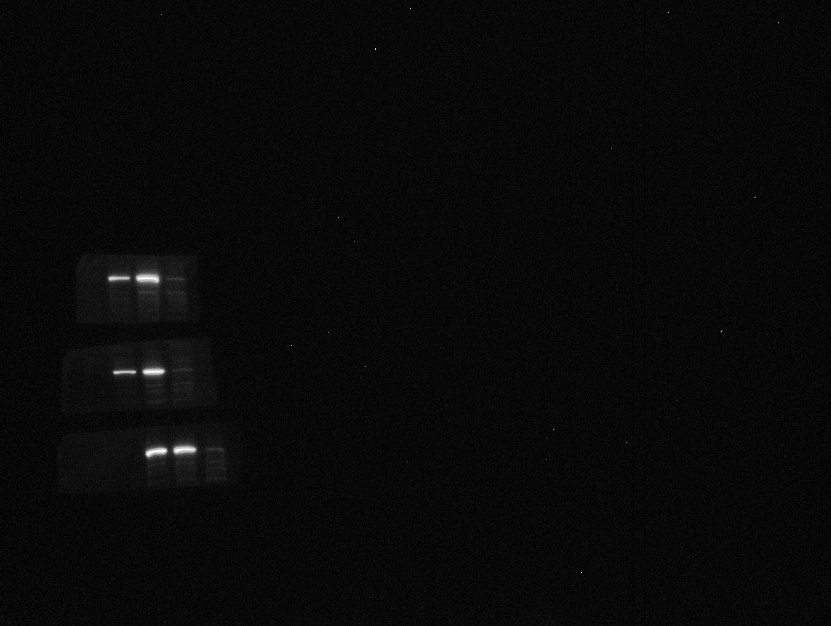

Supplement: Figure 7—source data 4. [file elife-88828-fig7-data4.zip › Figure 7-source data 4/ROCK/2022-02-16_13-41-57 Shrock_1_16bit.png]

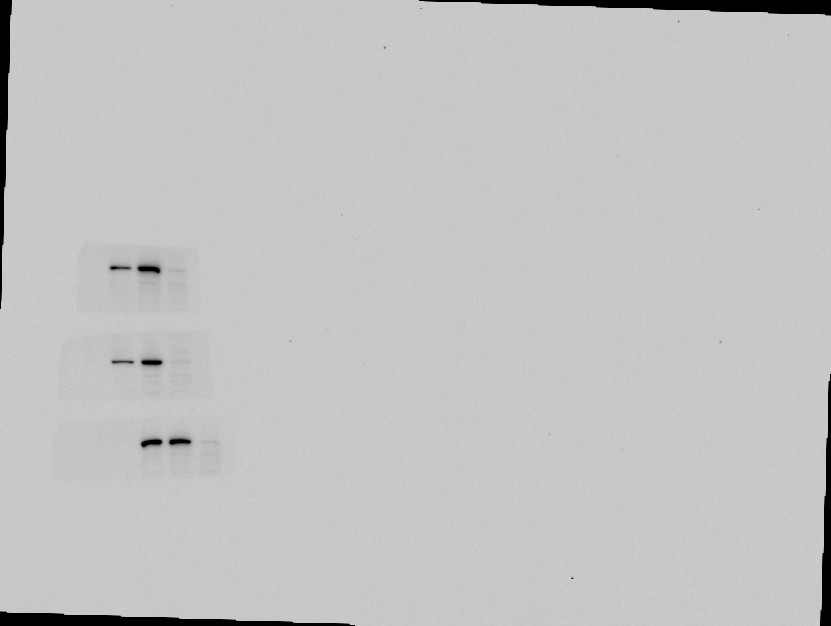

Supplement: Figure 7—source data 4. [file elife-88828-fig7-data4.zip › Figure 7-source data 4/ROCK/2022-02-16_13-41-57 Shrock_1_16bit.png-Deuteranope.tif]

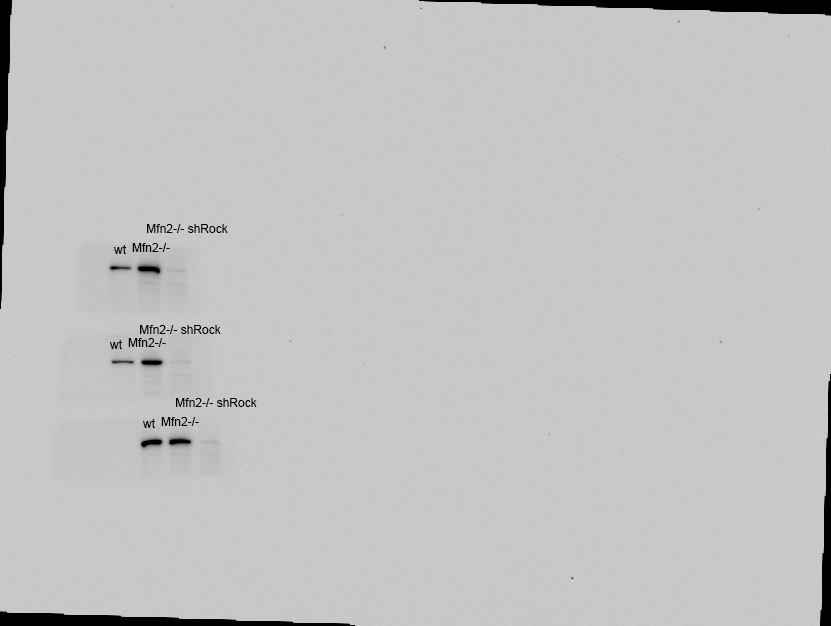

Supplement: Figure 7—source data 4. [file elife-88828-fig7-data4.zip › Figure 7-source data 4/ROCK/2022-02-16_13-41-57 Shrock_1_16bit.png-label.tif]

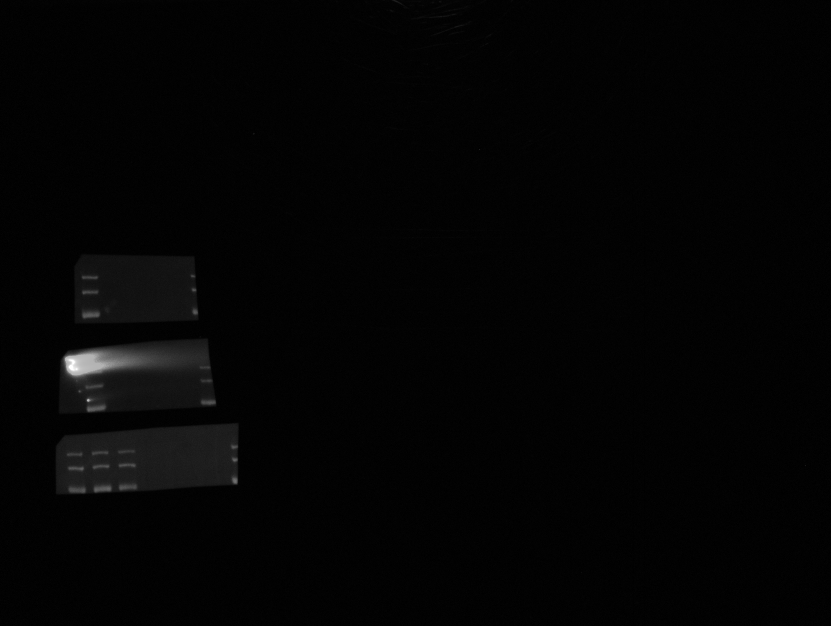

Supplement: Figure 7—source data 4. [file elife-88828-fig7-data4.zip › Figure 7-source data 4/ROCK/2022-02-16_13-41-57 Shrock_2_16bit.png]

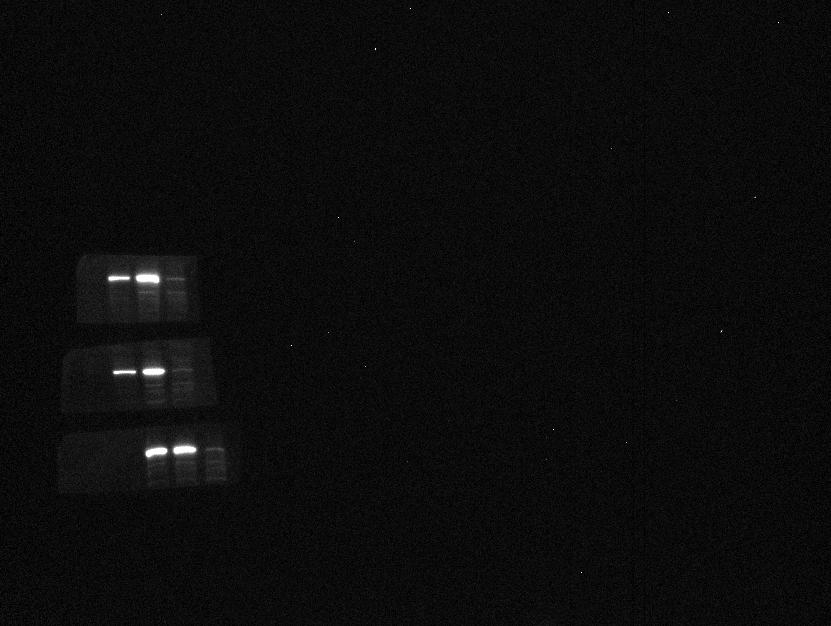

Supplement: Figure 7—source data 4. [file elife-88828-fig7-data4.zip › Figure 7-source data 4/ROCK/2022-02-16_13-41-57 Shrock_8bit.png]

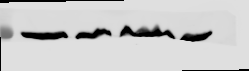

Supplement: Figure 7—source data 4. [file elife-88828-fig7-data4.zip › Figure 7-source data 4/TUBULIN-shMLCK/0000730_01_700.TIF-Deuteranope.tif]

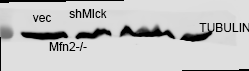

Supplement: Figure 7—source data 4. [file elife-88828-fig7-data4.zip › Figure 7-source data 4/TUBULIN-shMLCK/0000730_01_700.TIF-label.tif]

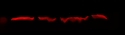

Supplement: Figure 7—source data 4. [file elife-88828-fig7-data4.zip › Figure 7-source data 4/TUBULIN-shMLCK/0000730_01_TH.jpg]

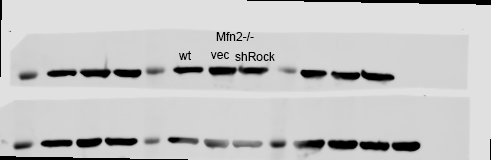

Supplement: Figure 7—source data 4. [file elife-88828-fig7-data4.zip › Figure 7-source data 4/TUBULIN-shROCK/0000737_01_700.TIF-label.tif]

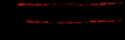

Supplement: Figure 7—source data 4. [file elife-88828-fig7-data4.zip › Figure 7-source data 4/TUBULIN-shROCK/0000737_01_TH.jpg]
